# Parallel detection and spatial mapping of large nuclear spin clusters

**DOI:** 10.1038/s41467-022-28935-z

**Published:** 2022-03-10

**Authors:** K. S. Cujia, K. Herb, J. Zopes, J. M. Abendroth, C. L. Degen

**Affiliations:** 1 Department of Physics, ETH Zurich, Otto Stern Weg 1, 8093 Zurich, Switzerland; 2 Quantum Center, ETH Zurich, 8093 Zurich, Switzerland; 3 Present Address: IT’IS Foundation, Zeughausstrasse 43, 8004 Zurich, Switzerland; 4 Present Address: Ansys Switzerland GmbH, Technoparkstrasse 1, 8005 Zurich, Switzerland

**Keywords:** NMR spectroscopy, Quantum metrology, Imaging techniques

## Abstract

Nuclear magnetic resonance imaging (MRI) at the atomic scale offers exciting prospects for determining the structure and function of individual molecules and proteins. Quantum defects in diamond have recently emerged as a promising platform towards reaching this goal, and allowed for the detection and localization of single nuclear spins under ambient conditions. Here, we present an efficient strategy for extending imaging to large nuclear spin clusters, fulfilling an important requirement towards a single-molecule MRI technique. Our method combines the concepts of weak quantum measurements, phase encoding and simulated annealing to detect three-dimensional positions from many nuclei in parallel. Detection is spatially selective, allowing us to probe nuclei at a chosen target radius while avoiding interference from strongly-coupled proximal nuclei. We demonstrate our strategy by imaging clusters containing more than 20 carbon-13 nuclear spins within a radius of 2.4 nm from single, near-surface nitrogen–vacancy centers at room temperature. The radius extrapolates to 5–6 nm for ^1^H. Beside taking an important step in nanoscale MRI, our experiment also provides an efficient tool for the characterization of large nuclear spin registers in the context of quantum simulators and quantum network nodes.

## Abstract (web summary)

Nuclear magnetic resonance imaging at the atomic scale has been limited to detection and localisation of single nuclear spins. Here, the authors extend imaging to large nuclear spin clusters in 3D by combining weak quantum measurements, phase encoding and simulated annealing.

## Introduction

Nuclear magnetic resonance (NMR) spectroscopy and magnetic resonance imaging (MRI) are powerful tools for molecular analysis and medical diagnostics. While conventional NMR operates on millimeter-sized samples containing large ensembles of molecules, much effort has been directed toward improving the resolution to the nanometer scale^1,2^ where the atomic structure could be analyzed at the level of individual molecules^3^. Such a “single-molecule MRI” technique would enable direct imaging of molecular structures with three-dimensional resolution and elemental specificity^1^ and the monitoring of chemical reactions and binding^4,5^. This capability could lead to many applications in biology, chemistry, and nanosciences, especially because MRI avoids important limitations of other structural techniques (like X-ray diffraction or electron microscopy) such as radiation damage and the need for ensemble averaging^6,7^.

Quantum sensors based on nitrogen-vacancy (NV) centers in diamond have recently generated exciting progress in micron-scale^8,9^ and nanoscale^10–12^ NMR spectroscopy. Early experiments have demonstrated the detection of single nuclear spins within the diamond crystal^13–15^ as well as of nanoscale films deposited on diamond surfaces^10–12,16^. A recent refinement of protocols has led to tremendous advances in sensitivity and spectral resolution^8,17,18^, allowing for the three-dimensional localization of individual nuclear spins^19–22^, spin pairs^23–25^, and the chemical fingerprinting of molecular ensembles with high spectral resolution^8,26^. Most recently, Abobeih et al.^27^ reported the milestone achievement of a complete mapping of a 27-nuclear-spin cluster at cryogenic temperatures.

To extend experiments to the imaging of single molecules, approaches are needed that are compatible with near-surface NV centers (≲5 nm) and preferably an ambient environment. Moreover, methods are required that can efficiently detect and precisely localize a large number of distant nuclear spins in parallel. While advanced strategies have been developed to solve the latter challenge of nuclear spin detection and localization^27–32^, many of these strategies require very long coherence times or a single-shot readout of the quantum sensor to reach adequate sensitivity and spectral resolution^27,33^. These conditions are difficult to realize with shallow defect centers at room temperature^34,35^.

In this work, we demonstrate a powerful method for the sensitive detection and spatial mapping of individual nuclei in large nuclear spins clusters. Our approach combines the concepts of weak quantum measurements^36,37^, phase encoding^21,22^, and simulated annealing^38,39^ to detect signals and extract precise three-dimensional distances from many nuclei in parallel. We further show that our detection is spatially selective, allowing us to probe nuclei at a chosen target radius while avoiding interference from strongly-coupled proximal nuclei. We demonstrate our strategy by mapping the ^13^C environment of two NV centers containing 20 and 29 nuclei, respectively. Because our experiments are performed on near-surface spin defects and at room temperature, they are compatible with the demanding environment of prospective single-molecule MRI investigations. Besides taking an important step in developing a single-molecule MRI platform, our experiment also provides an efficient tool for the characterization of large qubit registers in the context of quantum simulators^40^, quantum network nodes^33,41,42^, and multi-qubit quantum processors^43,44^.

## Results

### Imaging concept

**Fig. 1:**
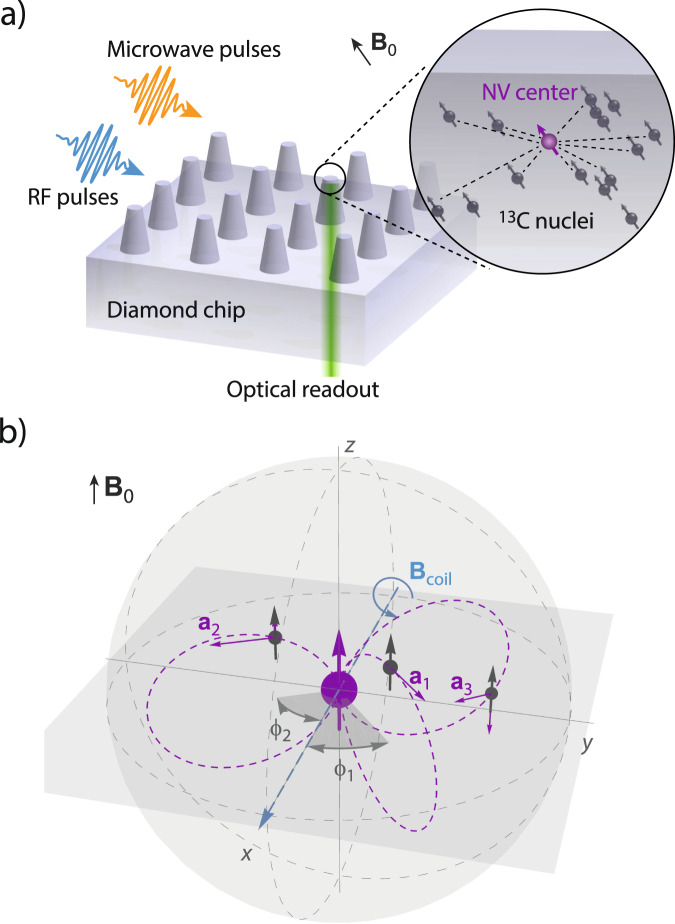
Concept of the nuclear spin mapping experiment. **a** We detect and image nuclear spins (black) surrounding a central electronic spin (purple). In our experiments, spins are embedded in a nanostructured diamond chip, manipulated by microwave and radio-frequency (RF) pulses, and detected by optical means. **b** Spatial imaging is enabled through the hyperfine field ${{{{{{{\bf{a}}}}}}}}=({a}_{\perp }\cos \phi ,{a}_{\perp }\sin \phi ,{a}_{| | })$, whose magnitude and direction strongly depend on the three-dimensional position **r** = (*r*, *ϑ*, *ϕ*). We determine the radius *r* and polar angle *ϑ* by measuring the parallel and transverse hyperfine components, *a*_∣∣_ = **a** ⋅ **e**_*z*_ and *a*_⊥_ = ∣**a** × **e**_*z*_∣^19,54^. The azimuth *ϕ* is equal to the phase of the nuclear precession^21,22^. **e**_*z*_ is a unit vector pointing along the electronic quantization axis and **B**_0_∣∣**e**_*z*_ is an external bias field. **B**_coil_∣∣**e**_*x*_ is the direction of the RF field.

Our concept and the experimental situation is sketched in Fig. 1a. We consider a central electronic spin surrounded by a group of nuclear spins whose three-dimensional locations we aim to determine. Here, both the electronic and nuclear spins are embedded in the solid matrix of a diamond crystal, but our concept is applicable to a general situation of a localized electronic spin^45–47^ and a nearby nuclear ensemble, including surface molecules^10,11,48^ or crystalline layers^49^. The electronic spin plays a dual role in our arrangement^30^: first, it acts as a local sensor for the weak magnetic fields produced by the nearby nuclei. Second, it generates a strong magnetic dipole field that we exploit for spatial imaging. In a reference frame where *z* is the common quantization axis (Fig. 1b), the dipole field is given by:

1 $${{{{{{{\bf{a}}}}}}}}/{\gamma }_{{{{{{{{\rm{n}}}}}}}}}=\frac{{\mu }_{0}\hslash {\gamma }_{{{{{{{{\rm{e}}}}}}}}}{m}_{S}}{4\pi {r}^{3}}\left[\frac{3{{{{{{{\bf{r}}}}}}}}({{{{{{{{\bf{e}}}}}}}}}_{z}\cdot {{{{{{{\bf{r}}}}}}}})}{{r}^{2}}-{{{{{{{{\bf{e}}}}}}}}}_{z}\right]\,,$$

where **a** is the hyperfine vector (see Fig. 1b), **r** = (*r*, *ϑ*, *ϕ*) are the polar coordinates of the nuclear spin relative to the electron spin situated at the origin, **e**_*z*_ is a unit vector along *z*, *m*_*S*_ is the magnetic quantum number of the electronic spin (${m}_{S}\in \left\{-1,0,1\right\}$ for the NV center). Because we will be detecting distant ^13^C spins, the Fermi contact interaction can be safely neglected^20,50^. Further, *μ*_0_ is the vacuum permeability, *ℏ* the reduced Planck constant, and *γ*_e_ and *γ*_n_ are the electronic and nuclear gyromagnetic ratios, respectively. Thus, by measuring the three components of the hyperfine vector **a**, the distance vector **r** can be directly inferred (up to an inversion symmetry at the origin), revealing a spin’s three-dimensional spatial location.

### Parallel signal acquisition

**Fig. 2:**
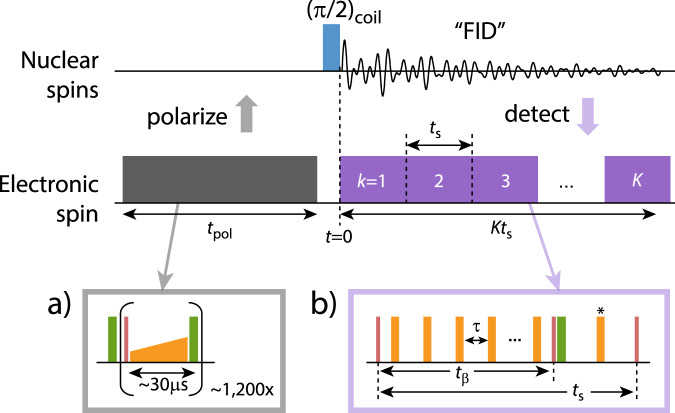
Weak-measurement protocol for detecting the free precession signal from many nuclei in parallel. We polarize the nuclear spins by a repeated NOVEL sequence (gray, inset **a**)^87,88^, initiate simultaneous precession of all nuclei by applying a *π*/2 pulse with an external RF coil (blue)^62^, and detect the precession by repeated sampling of the transverse nuclear magnetization (purple)^17,18,89^. *t*_pol_ is the polarization time, *t*_s_ is the sampling time and *K* is the number of samples. Each weak-measurement read-out block (inset **b**) consists of a Carr–Purcell–Meiboom–Gill (CPMG) pulse train^90–92^ of 4−24 equidistant *π* pulses (orange) separated by a delay time *τ* = 1/(2*γ*_n_*B*_0_), followed by an optical read-out pulse (green). Red blocks are *π*/2 pulses. The duration of the CPMG sequence defines the interaction time *t*_*β*_. An additional *π* pulse (*) is used to average over the electronic *m*_*S*_ = 0, −1 states. See the Supplementary Information for the full-timing diagram and experimental parameters.

While this scheme of three-dimensional localization has been demonstrated on individual nuclear spins^19–22^, the principal challenge lies in extending these experiments to large numbers of nuclei. We address this challenge by exploiting the principle of weak quantum measurements^36,51^, which closely resembles the detection of a free induction decay (FID) signal in canonical Fourier NMR spectroscopy. Figure 2 introduces our experimental protocol, consisting of a polarization, excitation, and read-out step. We begin by hyperpolarizing nuclear spins through a polarization transfer from the optically-aligned electronic spin (Fig. 2a). This initial step, when applied repetitively and for a sufficiently long time, leads to a volume of near-fully polarized nuclei around the central electronic spin^52^. We then excite all nuclei simultaneously using a broad-band *π*/2 pulse and detect the free nuclear precession signal by sampling the transverse nuclear magnetization using weak measurements^36^. The procedure yields an FID signal of the form:

2 $$x(t)=\mathop{\sum }\limits_{i=1}^{n}A({\beta }_{i}){e}^{-{{\Gamma }}({\beta }_{i})t}\cos [{\omega }_{i}t+{\phi }_{i}]\,,$$

where *n* is the number of nuclear spins. Further, *A*(*β*_*i*_) is the probability amplitude^17^, Γ(*β*_*i*_) the dephasing rate, *ω*_*i*_ the precession frequency, and *ϕ*_*i*_ the initial phase of the signal belonging to the *i*’th nucleus. The parameter:

3 $$\begin{array}{r}{\beta }_{i}=\frac{{a}_{\perp ,i}{t}_{\beta }}{\pi }\end{array}$$

is a “measurement gain” parameter that is proportional to the hyperfine coupling constant *a*_⊥,*i*_ multiplied by the interaction time *t*_*β*_ of the ac detection (Fig. 2b). The parameter *β*_*i*_, discussed in Section V, plays an important role in single-spin FID detection as it governs the balance between signal gain and quantum back-action^36,37,53^. We sample the FID at instances *t* = *kt*_s_, where *t*_s_ is the sampling time, *k* = 1 $\ldots$ *K*, and where *K* is the number of points in the FID trace (Fig. 2).

### Hyperfine parameters

We next show that an FID trace described by Eq. (2) contains all the information needed to reconstruct the hyperfine vectors **a**_*i*_, and hence, the three-dimensional locations **r**_*i*_ of the nuclear spins. First, the parallel components *a*_∣∣,*i*_ (see Fig. 1b) can be determined from the spectral positions of the nuclear resonances, given by the free precession frequencies:

4 $$\begin{array}{rc}{\omega }_{i}&=\frac{1}{2}\left({\omega }_{0}+\sqrt{{({\omega }_{0}+{a}_{| | ,i})}^{2}+{a}_{\perp ,i}^{2}}\right)\approx {\omega }_{0}+\frac{1}{2}{a}_{| | ,i}\,,\end{array}$$

where the approximation is for small *a*_∣∣,*i*_, *a*_⊥,*i*_ ≪ *ω*_0_ (fulfilled in our experiments but not required for the analysis) and where *ω*_0_ = −*γ*_n_*B*_0_, with *B*_0_∣∣**e**_*z*_ being the external magnetic bias field^54^.

The amplitudes *A*(*β*_*i*_) and decay rates Γ(*β*_*i*_) encode information about the perpendicular components *a*_⊥,*i*_ (See Supplementary Materials accompanying this manuscript):

5a $$A({\beta }_{i})=\frac{1}{2}{p}_{{{{{{{{\rm{0}}}}}}}},i}\sin \left({\beta }_{i}\right)\approx \frac{{p}_{{{{{{{{\rm{0}}}}}}}}}{a}_{\perp ,i}{t}_{\beta }}{2\pi }\,,$$

5b $${{\Gamma }}({\beta }_{i})=\frac{{a}_{\perp ,i}^{2}{t}_{\beta }^{2}}{4{t}_{{{{{{{{\rm{s}}}}}}}}}{\pi }^{2}}+\frac{{a}_{| | ,i}^{2}{t}_{\ell }^{2}}{2{t}_{{{{{{{{\rm{s}}}}}}}}}}+\frac{1}{{T}_{2,{{{{{{{\rm{n}}}}}}}}}^{* }}\,,$$

where *p*_0,*i*_ is the initial polarization of the *i*’th nuclear spin. Note that the *p*_0,*i*_ also contain any pulse errors and other imperfections of the pulse sequence, and therefore rather reflect pre-scaling factors and a lower bound for the nuclear polarization. The dephasing rate Γ(*β*_*i*_) is influenced by three effects: a measurement-induced dephasing proportional to ${a}_{\perp ,i}^{2}{t}_{\beta }^{2}$ due to quantum back-action^36^. (ii) An additional decay rate proportional to ${a}_{| | ,i}^{2}{t}_{\ell }^{2}$ that is specific to the stochastic optical read-out process of the NV center with effective duration *t*_*ℓ*_^36^. (iii) An intrinsic ${T}_{2,i}^{* }$ decay that accounts for all dephasing mechanisms not associated with the read-out process, such as spin-lattice effects or unresolved nuclear-nuclear couplings.

Finally, the azimuth *ϕ*_*i*_ is encoded in the complex phase of the nuclear FID signal. Because we initiate the FID by applying a *π*/2 pulse with an external RF coil, all nuclei are rotated around a common laboratory-frame axis and start precession with the same phase. By contrast, the ac detection of the FID is phase-sensitive with respect to each nucleus’ individual hyperfine field. As a consequence, the phase *ϕ*_*i*_ is equal to the spatial angle between the coil and hyperfine axes in the laboratory frame (Fig. 1b)^21,22^. Analysis of the complex FID signal therefore directly reveals the desired azimuth *ϕ*_*i*_.

In the following, we will make three important assumptions that are necessary for keeping the maximum likelihood fitting of spectra tractable. First, we assume that all nuclei carry approximately the same polarization, *p*_0,*i*_ ≈ *p*_0_. Because we repeat the polarization transfer process for typically >10^3^ cycles (i.e., longer than the FID duration), we expect that all nuclei within the sensitive radius become close to fully polarized. The polarization level may be slightly reduced for spin pairs^55^ or due to residual spin diffusion, however, these effects are small for our dilute ^13^C concentration. Our assumption of nearly full polarization is consistent with the observation that spectra show little change in peak intensities once the number of cycles is increased beyond ≳10^3^ ^36^. Similar saturation behavior is suggested by ref. ^52^. Second, we treat ${T}_{2,i}^{* }\approx {T}_{2}^{* }$ as a global parameter. Although this assumption is likely wrong in general, the role of ${T}_{2}^{* }$ here is that of an upper bound in the FID decay. Because the FID is for most spins dominated by the measurement-induced dephasing (i) and (ii), our method is not very sensitive to variations in ${T}_{2}^{* }$. We find that our fit results for ${T}_{2}^{* }$ are similar to those expected from the drift in the bias field, suggesting that our linewidths are limited by external field stability. Third, we will neglect nuclear-nuclear couplings, discussed further below.

### Sensitive slice

The magnitude of the FID signal strongly depends on a spin’s three-dimensional position **r**, because of the position dependence of the hyperfine interaction. We can capture the spatial dependence by calculating a sensitivity function ${{{{{{{\mathcal{S}}}}}}}}({{{{{{{\bf{r}}}}}}}})$ that quantifies the signal contribution as a function of spin location **r**. The sensitivity function is expressed as a signal-to-noise ratio and given by:

6 $${{{{{{{\mathcal{S}}}}}}}}({{{{{{{\bf{r}}}}}}}})\equiv {{{{{{{\mathcal{S}}}}}}}}(\beta ({{{{{{{\bf{r}}}}}}}}))=\frac{A(\beta )}{{{\Gamma }}(\beta ){t}_{{{{{{{{\rm{s}}}}}}}}}\sqrt{K}}\frac{(1-{e}^{-{{\Gamma }}(\beta )K{t}_{{{{{{{{\rm{s}}}}}}}}}})}{\sqrt{{t}_{{{{{{{{\rm{pol}}}}}}}}}+K{t}_{{{{{{{{\rm{s}}}}}}}}}}}$$

where *β* = *a*_⊥_*t*_*β*_/*π* (Eq. (3)) encodes the spatial position (via the hyperfine parameter *a*_⊥_), and where *K*, *t*_s_, *t*_*β*_, and *t*_pol_ are experimental parameters defined in Fig. 2.

**Fig. 3:**
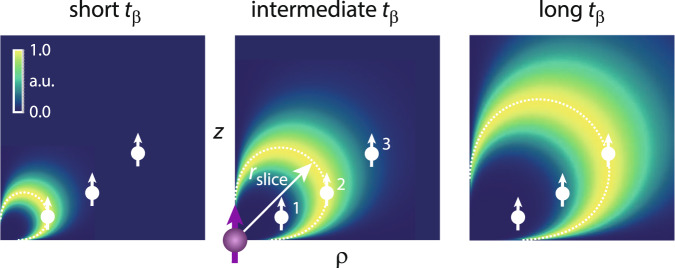
Simulation of the sensitive slice ${{{{{{{\mathcal{S}}}}}}}}({{{{{{{\bf{r}}}}}}}})$. *ρz*-plot of the sensitive slice for different interaction times *t*_*β*_, where $z=r\cos \vartheta$ and $\rho =r\sin \vartheta$ are vertical and radial distance, respectively. Bright shading color codes the signal-to-noise ratio. By varying *t*_*β*_ the sensitive slice can be tuned to spins in close (1), intermediate (2), or far (3) distance from the central electronic spin (panels from left to right). The dashed contour is the *a*_⊥_ isoline where *β* = *β*^(opt)^. *r*_slice_ is the characteristic radius of a slice. Plots assume *t*_*ℓ*_ = 0 and ${({T}_{2,{{{{{{{\rm{n}}}}}}}}}^{* })}^{-1}=0$.

Figure 3 plots ${{{{{{{\mathcal{S}}}}}}}}({{{{{{{\bf{r}}}}}}}})$ as a function of vertical and radial distance to the central electronic spin. Interestingly, the sensitivity does not monotonically decay with distance, as might be expected from the *a*_⊥_ ∝ *r*^−3^ scaling of the hyperfine interaction. Rather, ${{{{{{{\mathcal{S}}}}}}}}$ is initially low, and increases with *r* until it reaches a maximum at a characteristic radius *r*_slice_ before showing the expected *r*^−3^ decay. The suppression of signal from close spins is a consequence of quantum back-action^36^: Because these spins are strongly coupled, their measurement strength parameter *β* is large, leading to a rapid signal decay 1/Γ(*β*) ∝ *β*^−2^ → 0 (Eq. (5b)). Conversely, distant spins with small *β* generate weak signals because *A*(*β*) → 0 (Eq. (5a)). Maximum sensitivity results at an intermediate value where the two effects are balanced,

7 $${\beta }^{({{{{{{{\rm{opt}}}}}}}})}\approx \frac{2}{\sqrt{K}}\,.$$

The optimum point of sensitivity is approximately reached when intrinsic and induced decay rates are commensurate, ${({T}_{2,{{{{{{{\rm{n}}}}}}}}}^{* })}^{-1}={\beta }^{2}/(4{t}_{{{{{{{{\rm{s}}}}}}}}})$ and when the FID record length is matched to the decay rate, *Kt*_s_ = 1/Γ.

As shown in Fig. 3, the points of maximum sensitivity are located along a contour of constant *a*_⊥_ = *πβ*^(opt)^/*t*_*β*_. We denote this contour as the “sensitive slice” associated with the interaction time *t*_*β*_. By varying *t*_*β*_, we can vary the radius of the sensitive slice *r*_slice_ and tune detection from close to distant nuclear spins (Fig. 3, left to right). Because *a*_⊥_ ∝ *r*^−3^, the radius of the sensitive slice scales as ${r}_{{{{{{{{\rm{slice}}}}}}}}}\propto {t}_{\beta }^{1/6}$. The spatial selectivity is an important feature of our method, since it allows us to selectively probe nuclear spins at a defined (far) distance from the central electronic spin while avoiding interference from strongly-coupled nuclei in close proximity. Further, by sweeping *t*_*β*_, we can collect FID traces from several sensitive slices and cover an extended spatial volume in the sample.

### Maximum likelihood estimation by simulated annealing

Armed with a protocol for measuring the signals and coupling constants from many nuclei in parallel, we develop a maximum likelihood protocol to extract the hyperfine parameters and position vectors from an FID trace (Eq. 2).

We begin by setting up a likelihood model. Assuming *n* spins are contributing to the signal, our model contains *M* = 3*n* + 3 unknown parameters, including the three hyperfine parameters *a*_∣∣,*i*_, *a*_⊥,*i*_, and *ϕ*_*i*_ for each spin *i* plus three additional, global parameters accounting for an initial polarization *p*_0_ and dephasing times ${T}_{2,{{{{{{{\rm{n}}}}}}}}}^{* }$ and *t*_*ℓ*_ [see Eq. (5)]. Note that because the number of spins *n* is a priori unknown, *M* is itself a free parameter. To proceed, we collect the unknown parameters in the parameter vector ${{{{{{{\boldsymbol{\theta }}}}}}}}=\left\{{\theta }_{m}\right\}$, where *m* = 1…*M*. Our goal is to balance goodness of the fit and model complexity by minimizing a cost function of the form:

8 $${{{{{{{\rm{IC}}}}}}}}=G({{{{{{{\boldsymbol{\theta }}}}}}}},{{{{{{{\bf{x}}}}}}}})+P(K,M)\,,$$

where *G*(***θ***, **x**) is a measure of the goodness of the fit, ${{{{{{{\bf{x}}}}}}}}=\left\{{x}_{k}\right\}$, where *k* = 1…*K*, is the set of measured data points, and *P*(*K*, *M*) is a penalty term to prevent over-fitting^56^. Eq. (8) is the generic form of a so-called information criterion (IC). In our likelihood framework, *G*(***θ***, **x**) can be expressed in terms of a negative likelihood function^57^:

9 $$G({{{{{{{\boldsymbol{\theta }}}}}}}},{{{{{{{\bf{x}}}}}}}})=-2\ln \left({{{{{{{\mathcal{L}}}}}}}}[{{{{{{{\boldsymbol{\theta }}}}}}}},{{{{{{{\bf{x}}}}}}}}]\right)=K\ln \left(\mathop{\sum }\limits_{k=1}^{K}{[{x}_{k}-{\tilde{x}}_{k}({{{{{{{\boldsymbol{\theta }}}}}}}})]}^{2}\right),$$

where the argument of the logarithm is the residual sum of squares. The function ${\tilde{x}}_{k}({{{{{{{\boldsymbol{\theta }}}}}}}})$ represents the estimated data points calculated from Eq. (2) using the parameter vector ***θ***. For the penalty term *P*(*K*, *M*), we choose the so-called weighted-average information criterion (WIC)^58^ that is a weighted average of the Akaike and Bayesian information criteria (AIC^59^ and BIC^60^, respectively, see (See Supplementary Materials accompanying this manuscript for definition). The advantage of the WIC is that it performs well regardless of sample size *K*.

Minimization of Eq. (8) is highly nontrivial, as the number of fit parameters is large and *M* is itself an unknown. In this work, we implement generalized simulated annealing (GSA)^38,39,61^ algorithm to address this challenge. GSA is well-posed in our case because it works well for the global optimization of complicated, multi-dimensional systems with large numbers of local minima. To further improve the GSA, we run the minimization over a large number of random starting configurations for ***θ***. Finally, once a best-estimate (lowest IC) set of parameters has been found, we compute the three-dimensional locations **r**_*i*_ of nuclei from the hyperfine vector **a**_*i*_ by inverting Eq. (1) (see Eqs. (10, 11) in the Methods).

### Experimental demonstration

We experimentally demonstrate our three-dimensional nuclear localization strategy by imaging the ^13^C environment of shallow NV centers in diamond. We focus on two NV centers in this work, labeled NV1 and NV2, out of five recorded datasets. The two NV centers are selected for favorable optical contrast and electron spin coherence times, but not for their ^13^C environment. Their shallow depth (~10 nm) is not important for this study except for demonstrating that our method is compatible with near-surface NV centers. We probe the NV centers at room temperature using non-resonant optical excitation and a single-photon counting module. Electronic and nuclear spins are manipulated via two arbitrary waveform generators connected to a separate microwave transmission line and RF micro-coil circuits, respectively^21,62^. Experiments use a bias field *B*_0_ ~ 200 mT aligned to within 1^∘^ of the NV symmetry axis (Fig. 1b). A description of diamond samples and the experimental setup is provided in the Methods section.

**Fig. 4:**
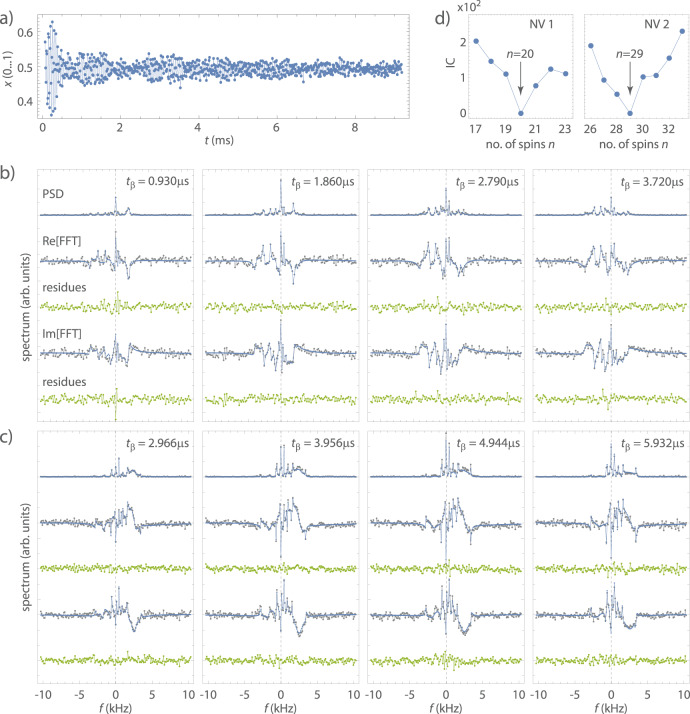
Experimental demonstration of ^13^C NMR spectroscopy of large spin clusters. **a** Example FID trace from NV2. Each data point reflects the probability amplitude *x*(*t*) from one read-out block, integrated over ~10^6^ repetitions of the full sequence (see Fig. 2). Measurement parameters are *t*_pol_ = 40 ms, *t*_s_ = 11.48 *μ*s, *t*_*β*_ = 4.944 *μ*s and *K* = 800, and total measurement time is 11 h. **b** Complex Fourier spectra of the ^13^C environment of NV1 for a series of interaction times *t*_*β*_. Shown are from top to bottom (vertically offset for clarity): power spectrum (PSD), real part of the complex spectrum (${{{{{{{\rm{Re}}}}}}}}[{{{{{{{\rm{FFT}}}}}}}}]$), fit residues for ${{{{{{{\rm{Re}}}}}}}}[{{{{{{{\rm{FFT}}}}}}}}]$, the imaginary part of the complex spectrum (${{{{{{{\rm{Im}}}}}}}}[{{{{{{{\rm{FFT}}}}}}}}]$), and fit residues for ${{{{{{{\rm{Im}}}}}}}}[{{{{{{{\rm{FFT}}}}}}}}]$. Blue traces are best fits (see text). The horizontal axis shows the spectral shift relative to the ^13^C Larmor frequency at 2.156 MHz calibrated using correlation spectroscopy^54^. The bias field is *B*_0_ = 201.29 mT. The PSD data of NV1 are the same as in ref. ^36^, Extended Data Fig. 5a. **c** Fourier spectra of the ^13^C environment of NV2. Bias field is *B*_0_ = 188.89 mT. **d** Cost function (IC, Eq. (8)) plotted as a function of the number of spins *n*. Best fits are obtained for *n* = 20 (NV1) and *n* = 29 (NV2). Since only differences in the cost function are meaningful, the minimum of the IC has been set to zero.

Figure 4a shows an example of an FID time trace from NV2 for *t*_*β*_ = 4.944 *μ*s, and Fig. 4b, c shows the complete dataset of ^13^C Fourier spectra obtained for both NV centers. For each NV center, we record four spectra with different values of the interaction time *t*_*β*_ to sample different radii of the sensitive slice and to add redundancy. For each dataset, we plot the power spectrum, the real and imaginary parts of the complex Fourier spectrum, as well as the fit residues. Clearly, the spectra show a rich peak structure, indicating that we are detecting a large number of ^13^C resonances.

To fit the spectra, we add the likelihood functions [Eq. 9] from all four spectra and minimize the total cost function [Eq. 8] using a single set of hyperfine parameters. We begin by randomly initializing each parameter, and then minimize the residues between the experimental and computed spectra using a GSA algorithm on a high-performance computer cluster^63^. To improve robustness, we fit the spectra, rather than the FID traces. We compute separate residues for real and imaginary parts of the complex spectrum as well as for their magnitude squared and minimize the sum of all residues. Additionally, we penalize configurations where the distance between any two ^13^C is less than one bond length. To accelerate the search for a global minimum, we repeat the procedure for a large number (~10^2^) of starting values randomly chosen from predefined parameter intervals. Finally, to determine the number of spins we run the minimization routine for different *n* and select the configuration with the smallest global IC value (Fig. 4d). Once the minimization has terminated and *n* has been determined, we perform bootstrapping on the final fit residues^64^ to obtain an estimate for the fit uncertainties for all parameters.

The calculated spectra for the most likely nuclear configurations are displayed as blue solid lines in Fig. 4b, c. For the two datasets, we find *n* = 20 for NV1 and *n* = 29 for NV2 (Fig. 4d). The large IC >10 of the next-best configurations *n* ± 1 indicates that our *n* are well-defined. (An IC >10 is equivalent to an evidence ratio of *e*^0.5IC^ > 10^2^, meaning that our estimated *n* is >10^2^ more likely than neighboring *n* ± 1.) This statistical finding is supported by the good agreement between fit and experimental data (gray dots) and the small residues (green), which are of the same order as the measurement noise. We have verified the calculated spectra by performing a full density matrix simulation using the final parameter set (Fig. S2). All fit results are collected in Tables S3 and S4.

**Fig. 5:**
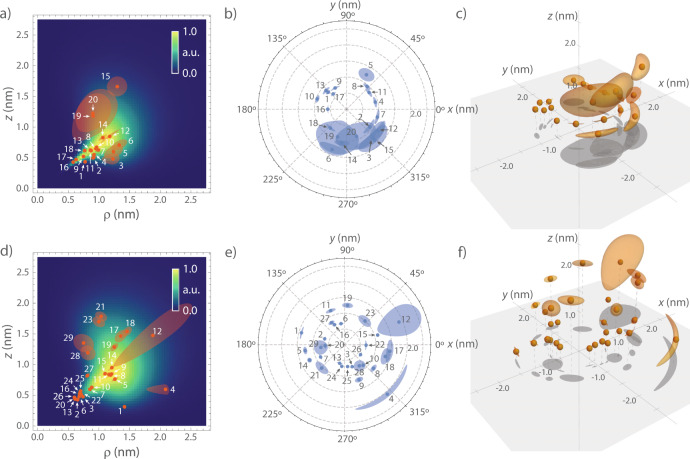
Visualizations of the three-dimensional nuclear spin mapping. **a** Fitted spatial locations of the ^13^C spins of NV1 (dots) shown in a *ρz*-plot. The central NV spin is located at the origin. Note that our method is ambiguous with respect to an inversion at the origin. Therefore, all nuclei are plotted in the upper hemisphere. Color coding reflects the combined sensitive slice [Eq. (6)] taking all *t*_*β*_ values as well as finite *t*_*ℓ*_ and ${T}_{2,{{{{{{{\rm{n}}}}}}}}}^{* }$ into account. **b** Polar plot showing the azimuth *ϕ* on the *xy*-plane. **c** Three-dimensional view of the nuclear positions. **d**–**f** Corresponding plots for the carbon spins of NV2. Uncertainties (±1 standard error) are shown as shaded areas or volumes, respectively, and are omitted if smaller than the data points. Index labels refer to Tables S3 and S4 in the Supplementary Information.

Figure 5 shows visualizations of the three-dimensional locations of nuclei. We find that nuclear positions are clustered between ca. *r* = 0.7–2.4 nm and ca. *ϑ* = 30–75^∘^. This clustering is a consequence of the spatial selectivity of our method: although the ^13^C nuclei are distributed randomly over the diamond lattice, only spins falling within the sensitive slice are picked up by the weak-measurement detection sequence. Proximal spins (*r* < 0.7 nm) could be detected by further reducing *t*_*β*_ or by employing techniques developed for strongly-coupled nuclei^19,20^. More distant spins (*r* > 2.4 nm) can be addressed by increasing *t*_*β*_, up to a limit set by the electronic coherence time *T*_2,e_.

We find that the combined sensitive slice for all measurements (color-coded in Fig. 5a, d), taking the *t*_*β*_ values of the four spectra, *t*_*ℓ*_ and ${T}_{2,{{{{{{{\rm{n}}}}}}}}}^{* }$ dephasing into account, agrees well with the extracted ^13^C positions. (The *t*_*ℓ*_ dephasing suppresses signal from nuclei with large *a*_∣∣_, leading to low sensitivity for spins near *ϑ* = 0^∘^ and 90^∘^). Figure 5 also clearly shows that the spatial precision of our method is highest and well below 1 Å for ^13^C’s that are located near the maximum of the sensitive slice, while the precision can be poor for spins located at the fringe of the slice.

The solution presented in Fig. 5 is a maximum likelihood estimate based on the assumptions of global values for the polarization and intrinsic dephasing as well as an absence of nuclear-nuclear couplings (see Section “Hyperfine parameters”). Our solution represents the three-dimensional nuclear spin configuration that has the highest likelihood, but there is no guarantee that it represents the “true” configuration of ^13^C nuclei on the diamond carbon lattice. This probabilistic aspect is a standard feature of large-scale structure determination^65^. For example, because our method does not account for nuclear couplings, it is possible that spin pairs or clusters of ^13^C are erroneously assigned a single-spin position. In addition, because of the inversion symmetry of the dipolar interaction [Eq. (1)], our method does not discriminate between spins lying in the upper and lower hemisphere. Looking forward, this ambiguity will be naturally lifted for outside molecules. Further, both issues can be alleviated by introducing additional spatial constraints, especially by measuring nuclear spin-spin distances using two-dimensional NMR spectroscopy^27^ (see Discussion).

To verify the three-dimensional nuclear configuration, we perform a set of basic statistical tests on the density, spatial distribution, and fit uncertainty of ^13^C positions. Comparing the volume uncertainties *δV* of the ^13^C (indicated by orange shading in Fig. 5c, e and tabulated in Supplementary Materials accompanying this manuscript.) with the volume per carbon atom in the diamond lattice (*V* = 5.67 Å^3^ ^66^), we find that 13 out of 20 spins (NV1) and 21 out of 29 spins (NV2) have *δV* < *V* and therefore likely represent single nuclei. Next, defining the volume of the sensitive region by the volume in space contributing 50% to the total signal^10^, we find sensitive volumes of *V* = 9.3 nm^3^ and 14.8 nm^3^ for NV1 and NV2, respectively. Considering an average density *ρ*(^13^C) = 1.94 nm^−3^ for ^13^C nuclei in diamond at natural isotope abundance (1.1%), the average number of ^13^C in the sensitive slices are 18.0 and 28.6, respectively, in good agreement with our experimental result. Further, a *χ*^2^ test for the angular distributions of the azimuth angles yields *p*-values well above the 5% level (54 and 70%, respectively), as expected for a random ^13^C distribution. Within these statistics, our experimental results are fully consistent with a stochastic distribution of ^13^C atoms around the NV centers. Finally, the *r* > 0.7 nm in retrospect justifies neglecting the Fermi contact interaction in our model^20,50^.

## Discussion

**Fig. 6:**
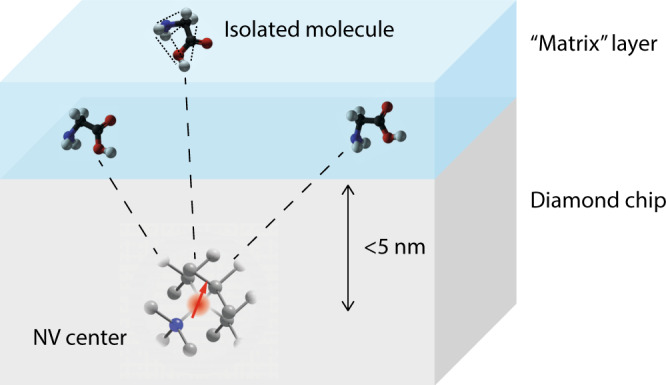
Proposed strategy for single-molecule MRI. A few molecules of interest are immobilized on a diamond surface containing <5-nm-deep NV centers. Molecules are protected by a spin-free “matrix” layer (blue shading). The global position and conformation of molecules are imaged by our weak-measurement localization scheme (dashed lines), while precise local distances between nuclei are measured using two-dimensional NMR (dotted lines). Computational analysis is used to extract three-dimensional atomic coordinates.

We conclude with a roadmap for extending the present experiment to samples outside of diamond, including individual molecules and complex spin structures (Fig. 6). Reaching this ambitious goal requires overcoming four central challenges:

(i) To image outside nuclear spins, near-surface NV centers are needed. Although our scheme is compatible with near-surface NV centers, their properties are known to degrade. The degradation manifests itself both in a reduced coherence time and reduced stability of the negative NV charge state. Recently, there has been remarkable progress in stabilizing very shallow NV centers. In particular, ref. ^67^ reports coherence times of *T*_2,e_ ~ 40 *μ*s for 5-nm-deep NV centers. In our experiments, we find similar values (*T*_2,e_ ~ 50 *μ*s for a 3.5-nm-deep NV center). Extrapolating the *r* ~ 2.4 nm for ^13^C (#12) in Fig. 5 using the $r\propto {\gamma }_{{{{{{{{\rm{n}}}}}}}}}^{1/3}{t}_{\beta }^{1/6}$ scaling of the sensitive radius, the above *T*_2,e_ implies a maximum radius of ca. 5–6 nm for single őr ^19^F nuclei (see Suppl. Fig. S5). This radius is compatible with the above shallow NV centers.
(ii) Single molecules must be immobilized and isolated on the diamond surface. This step can be achieved using surface functionalization^5,48,68^. To further protect the molecules and inhibit diffusion, molecules may be embedded in a spin-free matrix layer (Fig. 6)^69^.
(iii) Nuclear spins in molecules will behave differently from internal ^13^C nuclei. In particular, nuclear spin interactions will likely dominate over the hyperfine interaction. Nuclear spin interactions can be mitigated by homo- and heteronuclear decoupling techniques^70^ and by isotope dilution^71^; however, they also are an important resource for structural information and a central element in our molecular imaging strategy, see next point.
(iv) Advanced spectroscopy and imaging techniques are needed that can efficiently retrieve the desired structure. A promising strategy is to combine the global distance information available by our scheme with local distance constraints obtained by solid-state NMR methods^27^ (dashed and dotted lines in Fig. 6). For example, two-dimensional spectroscopy^9,25,72^ and double-echo techniques^27,73^ can be conveniently integrated with our protocol, because of its similarity to conventional FID detection. Additionally, efficient computational methods will be required to speed up data analysis and optimally extract the desired spatial information, including, for example, sparse sampling^29,74^, gradient Monte Carlo^75^, and machine learning techniques^76–78^.

By demonstrating three-dimensional mapping of nuclear spins in ambient conditions, our work takes an important step forward towards the ambitious goal of single-molecule MRI. Looking beyond the milestone of structural imaging, the technique could be extended to study chemical binding and chemical surface reactions at the single-molecule level. For example, in a biological context, surface NMR may allow investigation of a priori unknown substrate binding sites in enzymes^79^, as well as conformational changes in biomolecules associated with allosteric regulation^80^, protein aggregation^81^, or aptamer-target recognition^82^. These applications are particularly appealing because NMR can provide complementary information to other surface-sensitive techniques, such as surface-enhanced Raman spectroscopy (SERS), photoelectron spectroscopies, and fluorescence microscopy.

Besides magnetic imaging of single spins, our work also provides interesting perspectives for the characterization of large qubit registers in quantum applications. For example, our method can be applied to efficiently map out the coupling network of quantum nodes built from a central electronic spin backed by a nuclear spin register^83,84^. Such quantum nodes are central elements in emerging optical^85^ or electronic^86^ quantum interconnects. Another application is a nuclear quantum simulation using an electronic qubit for initialization and readout^40^. Finally, our parallel measurement protocol could provide a rapid means for calibrating cross-talk in superconducting qubit architectures^43,44^.

## Methods

### Diamond samples

Two single-crystal diamond plates were used for experiments. Both sample A (NV1) and sample B (NV2) were electronic-grade, natural abundance (1.1% ^13^C) diamond membranes. NV centers were created by ^15^N^+^ ion implantation at an energy of 5 keV and doses of 5 × 10^11^ cm^−2^ and 4 × 10^10^ cm^−2^ for samples A and B, respectively. Samples were subsequently annealed at 850 °C to form NV centers. We chose the ^15^N species to discriminate implanted NV centers from native (^14^N) NV centers. Both samples were cleaned in a 1:1:1 mixture of H_2_SO_4_:HNO_3_:HClO_4_ and baked at 465 °C in the air before mounting them in the setup. Whenever organic contamination was spotted, samples were cleaned in a 2:1 mixture of H_2_SO_4_:H_2_O_2_ (Piranha). We etched nano-pillars into the membrane surfaces to increase the photon collection efficiency. The continuous wave (CW) photon count rate was 250–500 kC/s.

### Experimental setup

Experiments were performed using a custom-built confocal microscope equipped with a green *λ* = 532 nm frequency-doubled Nd:YAG excitation laser (CNI Laser MSL-FN-532nm) and a 630−800 nm detection path using a single-photon avalanche photodiode (APD, Pelkin Elmer SPCM-AQR Series). Optical pulses were generated by an acousto-optic modulator (AOM, Crystal Technology 3200-144) in a double-pass configuration, and gating of arriving photons was realized by time-tagging (NI-PCIe-6363) and software binning of photon counts. Typical laser excitation powers were on the order of 100 *μ*W.

We synthesized microwave pulses for manipulating the electronic spin using an arbitrary waveform generator (AWG, Tektronix AWG5012C) and up-converted them to ~2.5 GHz using a local oscillator (Hittite HMC-T2100) and a quadrature mixer (Marki microwave IQ1545). Pulses were subsequently amplified (Gigatronics GT-1000A) prior to delivery to the NV center using a coplanar waveguide (CPW) photo-lithographically defined on a quartz cover slip. The transmission line was terminated on an external 50 Ω load (Meca 490-2). We synthesized radio-frequency (RF) pulses for nuclear spin manipulation using an AWG (National Instruments PCI-5421) and subsequently amplified them (Mini-Circuits LZY-22+). The pulses were transmitted using a planar micro-coil connected in series with a 50 Ω termination (Meca 697-30-1). The measured micro-coil inductance was *L* = 0.77 *μ*H. The 50 Ω termination increased the rf-circuit bandwidth (*Q* = *L*/*R*) at the expense of efficiency (most power was dissipated in the load). The micro-coil circuit had a 3-dB-bandwidth of ~19 MHz. ^13^C Rabi frequencies were typically around 25 kHz. A layout of the micro-coil arrangement is given in ref. ^62^.

We used a cylindrical samarium-cobalt permanent magnet (TC-SmCo, reversible temperature coefficient 0.001%/^°^C) to create a bias field *B*_0_ ~ 190 mT at the NV center location. To align *B*_0_ with the NV symmetry axis, we adjusted the relative location of the permanent magnet by fitting it to a set of electron paramagnetic resonance (EPR) lines recorded at different magnet locations and subsequently by maximizing the CW photon count rate.

### Tracking of magnetic field drifts

The net magnetic bias field drifted by typically a few *μ*T, leading to variations in the EPR frequency of order ±100 kHz and variations in the ^13^C Larmor frequency of order ±50 Hz. This limited the observed ^13^C linewidths to ~100 Hz. Field drifts were the dominating source of line broadening for the ^13^C NMR. We continuously tracked and logged magnetic field drifts by measuring the EPR resonance of the NV center and periodically readjusted the microwave excitation frequency during the course of the experiment.

### Hyperfine vector **a** and position **r**

The radius *r* and polar angle *ϑ* are computed from the parallel and transverse hyperfine parameters *a*_∣∣_ and *a*_⊥_ as follows (see ref. ^54^, Eq. S28 and S29):

10 $$\vartheta =\arctan \left\{\frac{1}{2}\left(-3\frac{{a}_{| | }}{{a}_{\perp }}+\sqrt{9\frac{{a}_{| | }^{2}}{{a}_{\perp }^{2}}+8}\right)\right\},$$

11 $$r={\left\{\frac{{\mu }_{0}{\gamma }_{{{{{{{{\rm{e}}}}}}}}}{\gamma }_{{{{{{{{\rm{n}}}}}}}}}\hslash (3{\cos }^{2}\vartheta -1)}{4\pi {a}_{| | }}\right\}}^{1/3}.$$

## Supplementary information

Supplementary Information

## Data Availability

The data that support the findings of this study are available from the corresponding author upon request.

## Code availability

The code that was used to perform the GSA minimization is available from the corresponding author upon request.
