# Supplementary material for: Parallel detection and spatial mapping of large nuclear spin clusters
Source: Nat Commun. 2022 Mar 10;13:1260. doi: 10.1038/s41467-022-28935-z (PMC8913684; doi:10.1038/s41467-022-28935-z)
Supplement: Supplementary file 1 — Supplementary Information [file 41467_2022_28935_MOESM1_ESM.pdf]

Supplementary Information: Parallel detection and spatial mapping of large nuclear spin clusters

K. S. Cujia[1,2], K. Herb[1], J. Zopes[1,3], J. M. Abendroth[1] and C. L. Degen[1,4]

[1]*Department of Physics, ETH Zurich, Switzerland.*
[2]*Present address: IT'IS foundation, Zeughausstrasse 43, 8004 Zurich, Switzerland.*
[3]*Present address: Ansys Switzerland GmbH, Technoparkstrasse 1, 8005 Zurich, Switzerland.*
[4]*Quantum Center, ETH Zurich, 8093 Zurich, Switzerland.*

1. BASICS OF WEAK MEASUREMENT SPECTROSCOPY

1.1. Single-spin free induction decay under periodic weak measurements

Weak measurements probe the transverse nuclear polarization (specifically, the $\langle \hat{I}_x \rangle$ component) linearly with the measurement strength β [1]. The measured free-induction decay (FID) signal for nuclear spin i as a function of time, that is, the measured transition probability for the spin sensor, has the form:

$$x_i\left(t\right) = \frac{1}{2} p_0 \sin\left(\beta\right) \cos\left(\omega_i t\right) e^{-\Gamma_i t} e^{-\left(t_\beta / T_{2,\mathrm{e}}\right)^a} \tag{S1}$$

where $\omega_i = \left| \frac{\gamma_n}{t} \int_0^t B\left(t'\right) dt' \right|$ is the average precession frequency of nuclear spin i. γ_n is the nuclear spin gyromagnetic ratio. For completeness, we have included the NV electronic spin coherence time $T_{2,\mathrm{e}}$, where a is an exponent which describes the dephasing rate of the spin sensor (exponential or Gaussian). Because in our experiments $t_\beta \ll T_{2,\mathrm{e}}$, sensor dephasing is not important for our study. p_0 describes the initial nuclear polarization.

Weak measurements simultaneously address all nuclear spins within the detector bandwidth of approximately t_β^{-1} [1]. Because the measurements are weak, $\beta \approx a_\perp t_\beta \ll 1$, we can neglect nuclear-nuclear interactions mediated by the sensor spin. For n nuclear spins, the total FID signal is thus the superposition of all individual contributions, leading to Eq. (2) in the main text.

1.2. Amplitude and decay rate

The evolution of a quantum state $\hat{\rho}_k$ describing a single nuclear spin subject to a series of sequential weak measurements of duration t_β at a repetition period t_s, is approximately given by [1]:

$$\hat{\rho}_k(t) \approx \hat{I}_e + \Big(\hat{I}_x \cos\left(\omega t\right) + \hat{I}_y \sin\left(\omega t\right)\Big) e^{-\Gamma_\beta t} + \mathcal{O}(\beta^2) \tag{S2}$$

where $t = k t_\mathrm{s}$ ($k = 1 \ldots K$), ω is the nuclear Larmor frequency, $\beta = a_\perp t_\beta / \pi$ is the measurement strength and $\Gamma_\beta = \beta^2/(4 t_\mathrm{s})$ is the measurement-induced dephasing due to quantum back-action.

Weak measurements map the expectation value $\langle \hat{I}_x \rangle$ of the nuclear-spin state, proportionally to the measurement strength β onto the optically-readable $\hat{S}_z$ sensor state [1]. The measured signal thus has an amplitude

$$A(\beta) = \frac{1}{2} p_0 \sin\left(\beta\right) \approx \frac{p_0 a_\perp t_\beta}{2\pi}. \tag{S3}$$

where p_0 is the initial polarization given by $\langle \hat{I}_x \rangle (t = 0)$. This is Eq. (5a) in the main text.

At the same time, a series of weak measurements leads to an exponential decay of the nuclear coherences $\hat{I}_x$ and $\hat{I}_y$, ultimately leading to a fully mixed state. Detailed analysis

of the spin-state projection shows [1] that the loss of coherence is, on average, $\beta^2/4$ per weak measurement, leading to an exponential decay rate $\Gamma_\beta = \beta^2/(4t_\mathrm{s})$. Further, owing to our off-resonant optical readout and re-initialization scheme, the NV center undergoes stochastic spin-state, electronic-state and potentially charge-state transitions during optical illumination [2, 3]. This leads to stochastic jumps in the hyperfine interaction experienced by the nuclear spin, leading to a "readout-induced" dephasing rate $\Gamma_\mathrm{optical} = \frac{\left(a_{||}t_\ell\right)^2}{2t_\mathrm{s}}$. Here, t_ℓ is an experimentally-determined parameter that roughly equals the time that the NV center spin is in an undefined m_S state during laser readout. Finally, nuclear spins are subject to intrinsic dephasing $\Gamma_0 = (T_{2,n}^*)^{-1}$ given by the intrinsic dephasing time $T_{2,n}^*$. The total decay rate is then the sum of all contributions:

$$\Gamma(\beta) = \frac{a_\perp^2 t_\beta^2}{4t_\mathrm{s}\pi^2} + \frac{a_{||}^2 t_\ell^2}{2t_\mathrm{s}} + \frac{1}{T_{2,\mathrm{n}}^*} \tag{S4}$$

This is Eq. (5b) in the main text.

1.3. Dynamic nuclear polarization (DNP)

DNP methods refer to the transfer of polarization from electron onto nuclear spins [?]. For this to occur, the involved energy scales must be commensurate. Thus, the main idea is to match the differing energy scales of the involved spin species by engineering their interaction, such that polarization transfer can take place. If the resulting timescales are faster than spin diffusion, repeated application can lead to hyperpolarization of the nuclear spin-bath [4, 5].

We employ a ramped nuclear-spin orientation via electron spin-locking (NOVEL) [6] sequence (Fig. S1), consisting on resonantly driving the electron-spin using a Rabi field with (variable) amplitude $2B_1$. To first order, the effective interaction has the form:

$$\begin{aligned}\hat{H} &= \left(\omega_n + \frac{a_{||}}{2} - \omega_1\right)\hat{I}_z + \frac{a_\perp}{4}\left(\hat{S}^+\hat{I}^- + \hat{S}^-\hat{I}^+\right) \\ &\approx \Delta\hat{I}_z + \frac{a_\perp}{2}\hat{I}_x\end{aligned} \tag{S5}$$

for each nuclear spin. $\hat{S}^\pm$, $\hat{I}^\pm$ are ladder operators for the electron (here in the x-basis) and nuclear spins, respectively. In our experiments, we work in the sub-space spanned by the electron-spin projections $m_S = \{0, -1\}$. Eq. S5 has the form of a canonical Rabi-drive, meaning that polarization transfer is driven by $a_\perp$ at a rate $\Omega = \sqrt{\Delta^2 + \left(a_\perp/2\right)^2}$. $\omega_n = \gamma_n B_0$ is the bare nuclear Larmor frequency and $\omega_1 = \sqrt{2}\gamma_n B_1$ is the Rabi frequency (the $\sqrt{2}$ factor accounts for the fact that we work with an $S = 1$ electron spin). $\Delta = \left(\omega_n + a_{||}/2\right) - \omega_1$ is the detuning. We assume the hyperfine couplings to be much faster than the timescales for spin-diffusion.

Since Δ is in principle different for each nuclear spin, the parallel hyperfine couplings $a_{||}$ introduce z-disorder in the nuclear-spin bath. This is an advantage since so-called dark

states [5, 7], which prevent complete bath polarization, are expected in systems without z-disorder. However, even in the presence of nuclear spins with identical ($a_{||}$,$a_{\perp}$) couplings, we expect a high degree of polarization upon repeated application of the NOVEL sequence.

2. SENSITIVE SLICE

We define the power signal-to-noise ratio (pSNR) as the ratio between the spectral peak amplitude and the standard deviation in a reference spectral region where there are no signals. For a single nuclear spin, the total signal after K weak measurements is given by [1]:

$$S = \frac{\epsilon C p_0 \sin(\beta)}{2\Gamma t_\mathrm{s}} \left(1 - e^{-\Gamma K t_\mathrm{s}}\right) \ , \tag{S6}$$

where C represents the total photon counts, ϵ the optical contrast, and $p_0\,\{0...1\}$ is the nuclear polarization. Two processes contribute to the total noise in a single weak measurement, a Bernoulli process resulting from quantum projection noise and a Poisson process associated with photon shot noise [8]. In our experiments, the photon shot noise greatly exceeds the quantum projection noise, and the noise variance is given by:

$$\sigma_x^2 = C\left(1 - \frac{\epsilon}{2}\right) \approx C \tag{S7}$$

where the last approximation is valid for small $\epsilon \ll 1$, which is usually the case in our experiments. The power SNR is given by:

$$\mathrm{pSNR} = \frac{S^2}{K\sigma_x^2} \approx \left(\frac{\epsilon C p_0 \sin(\beta)}{2\Gamma t_\mathrm{s}} \left(1 - e^{-\Gamma K t_\mathrm{s}}\right)\right)^2 \frac{1}{KC} \ . \tag{S8}$$

To obtain the amplitude SNR per unit time, we divide by the duration $t_\mathrm{m} = t_\mathrm{pol} + K t_\mathrm{s}$ of one complete measurement cycle and take the square root:

$$\mathrm{SNR}_0 = \sqrt{\frac{\mathrm{pSNR}}{t_\mathrm{m}}} \approx \frac{\epsilon\sqrt{C} p_0 \sin(\beta)}{2\Gamma\sqrt{K} t_\mathrm{s}} \frac{\left(1 - e^{-\Gamma K t_\mathrm{s}}\right)}{\sqrt{t_\mathrm{m}}} \ . \tag{S9}$$

We now define the sensitivity function $\mathcal{S}(\beta)$ by the amplitude SNR expressed as a function of β, dropping the count rate factor $\epsilon\sqrt{C}$ and taking the approximation $\sin(\beta) \approx \beta$:

$$\mathcal{S}(\beta) = \frac{\mathrm{SNR}_0}{\epsilon\sqrt{C}} \overset{\beta\ll 1}{\approx} \frac{p_0 \beta}{2\Gamma\sqrt{K} t_\mathrm{s}} \frac{\left(1 - e^{-\Gamma K t_\mathrm{s}}\right)}{\sqrt{t_\mathrm{m}}} \ . \tag{S10}$$

This equation corresponds to Eq. (6) in the main manuscript. Eq. S10 is maximized in the regions of large $a_\perp$ and vanishing $a_{||}$. In such case $\Gamma_\mathrm{optical} = 0$, and we can rewrite

$$\mathcal{S}(\beta) \propto \frac{1}{\sqrt{1 + \Gamma_0/\Gamma_\beta}} \frac{1}{\sqrt{\Gamma K t_\mathrm{s}}} \left(1 - e^{-\Gamma K t_\mathrm{s}}\right) \ , \tag{S11}$$

where we have set $p_0 = 1$ for simplicity. Eq. S11 reaches a maximum $\mathcal{S}_{\text{max}}$ at $\Gamma K t_{\text{s}} \approx 2\pi/5$ when $\Gamma_0 \to 0$. For finite Γ_0, an optimum point is reached when the intrinsic and induced decay rates are commensurate [1], *i.e.*, $\Gamma_0 \approx \Gamma_\beta$ and thus $\Gamma \approx 2\Gamma_\beta = \beta^2/(2t_{\text{s}}) = 2\Gamma_0$, meaning that:

$$\beta^{(\text{opt})} = 2\sqrt{\Gamma_0 t_{\text{s}}}\ . \tag{S12}$$

Since $\beta = \left(a_\perp/\pi\right) t_\beta$ and $a_\perp \propto r^{-3}$, assuming short sensor readout times $t_{\text{s}} \approx t_\beta$ the sensitivity radius at $\Gamma_0 \approx \Gamma_\beta$ scales as $r \propto t_\beta^{1/6}$. Specifically, we obtain:

$$r = \left[\left(\frac{a_0}{2\pi}\right)^2 \frac{t_\beta}{\Gamma_0}\right]^{1/6}\ , \tag{S13}$$

where $a_0 = \hbar\mu_0\gamma_n\gamma_e \left(3\cos\left(\theta_m\right)\sin\left(\theta_m\right)\right)/\left(4\pi\right)$ and $\theta_m = 54.7°$ is the magic-angle. $\hbar$ is the reduced Planck constant and μ_0 the free-space permeability. To maximize signal acquisition (*i.e.* Kt_{s}) while minimizing decay, we set $\Gamma_0 = \Gamma_\beta$ in Eq. S11 and numerically find the point at which the sensitivity has decayed by $1/e$, *i.e.* solve $\mathcal{S}\left(\beta^{(\text{opt})}\right) = \left(1 - e^{-1}\right)\mathcal{S}_{\text{max}}$ to find $\Gamma_0 K t_{\text{s}} \approx 3.06 \approx 0.5\pi\left(3+\pi\right)$, and thus:

$$K t_{\text{s}} \approx K t_\beta \approx \frac{3+\pi}{2\Gamma_0}\ . \tag{S14}$$

To verify Eqs. S13 and S14 we have numerically maximized Eq. S10 as a function of β and evaluated $\mathcal{S}$, r and Γ_0/Γ_β at β_{max} for different values of K and $t_{\text{s}} \approx t_\beta$. We find good agreement with our analytical approximations.

To compute the "combined" sensitive slices plotted in Fig. 5(a,d) of the main manuscript we evaluate the sensitivity function $\mathcal{S}\left(\beta\right)$ for each t_β value and take the maximum $\max\left[\mathcal{S}\left(\beta_i\right)\right]$ at each $\left(\rho, z\right)$ value.

3. MAXIMUM LIKELIHOOD ESTIMATION BY GENERALIZED SIMULATED ANNEALING

3.1. Information criteria

The selection of appropriate approximate models is critical for statistical inference out of experimental data. A very general methodology for model selection and parameter estimation is the use of information criteria and likelihood concepts [9]. When applying information criteria for model selection one aims to measure the distance or information, which in turn can be linked to the concept of entropy maximization, between two models [9].

From a simplified perspective, the idea is to balance the goodness of fit and the complexity of a model using a cost function of the form

$$\text{IC} = G\left(\sigma^2\right) + P\left(K, M\right)\ , \tag{S15}$$

where $G\left(\sigma^2\right)$ accounts for the goodness of fit and depends on an unknown variance estimator σ^2 of the fit residues. To minimize notation overhead, in the following we will refer to estimators of any quantity (*e.g.* variance, standard deviation, mean) simply with a letter. $P\left(K, M\right)$ can be regarded as a penalty which depends on the sample size K and the number of parameters M. In a likelihood framework, the goodness of fit can be expressed in terms of a negative likelihood function [9],

$$G\left(\sigma^2\right) = -2\ln\left(\mathcal{L}(\boldsymbol{\theta})\right) \ , \tag{S16}$$

where $\mathcal{L}(\boldsymbol{\theta})$ is the likelihood of the candidate model given the data, evaluated at the maximum likelihood estimate of the model parameters $\boldsymbol{\theta}$. Computing ΔG between two models is a measure known as the Kullback-Leibler information [9]. In isolation, Eqs. S15 and S16 are meaningless; only differences in their values when calculated using different models are useful. Different choices of $P\left(K, M\right)$ have been proposed, being the Akaike Information Criteria (AIC) [10] and the Bayesian Information Criteria (BIC) [11] the most common ones:

$$P_{\text{AIC}} = 2M + \frac{2M\left(M+1\right)}{K-M-1} = \frac{2MK}{K-M-1} \ , \tag{S17}$$

$$P_{\text{BIC}} = M\ln\left(K\right) \ , \tag{S18}$$

where M is the total number of estimated parameters in the model and K is the sample size. The last term in Eq. S17 is a correction introduced to compensate the AIC tendency to overfit for finite samples [12]. BIC has a strong penalty, and therefore can lead to underfitting in large samples [13]. The Weighted Average Information Criteria (WIC) [14] provides a solution which performs well, independent of the sample size:

$$P_{\text{WIC}} = \frac{P_{\text{AIC}}^2 + P_{\text{BIC}}^2}{P_{\text{AIC}} + P_{\text{BIC}}} \ . \tag{S19}$$

We use P_{WIC} as the the penalty term $P(K, M)$ in Eq. (8) of the main manuscript.

In the limit of large sample sizes $K \gg 1$, and assuming normally-distributed residues (errors) with a constant variance, the negative log-likelihood in Eq. S16 becomes (up to a constant term) [10]:

$$-2\ln\left(\mathcal{L}(\boldsymbol{\theta})\right) \approx K\ln\left(\sigma^2\right) \ , \tag{S20}$$

where σ^2 is the variance estimator for the true variance σ_0^2 of the residues. Note that the variance estimator is itself a function which depends on the data and the model parameters. For least-squares estimation, the maximum-likelihood estimator for the variance $\sigma^2 = \Sigma/K$, where Σ is the residual sum of squares (RSS), is usually used. However, this is a biased estimator. Instead, using the sample variance:

$$s^2 = \frac{\Sigma}{K-M-1} = \frac{\sum_{k=1}^{K}\epsilon_k^2}{K-M-1} \ , \tag{S21}$$

where ϵ_k are the individual residues, provides an unbiased estimator which contributes an additional penalty term and further avoids overfitting [13, 15].

We now derive Eq. S20 for our specific FID models. Assume a random variable x whose probability distribution $f_0(x;\boldsymbol{\theta})$ is defined on the parameters $\boldsymbol{\theta} = \{\theta_m\}$, where $m = 1 \ldots M$ and M is the number of fit parameters. A set of observations $\mathbf{x} = \{x_k\}$, where $k = 1 \ldots K$, can be regarded as K samples of the distribution $f_0(x;\boldsymbol{\theta})$. In our experiment, $\boldsymbol{\theta}$ are the three hyperfine parameters for each spin plus the three global parameters p_0, $T^*_{2,\mathrm{n}}$ and t_ℓ (see main manuscript), and $\mathbf{x}$ is the experimental FID trace. Assuming a model $f(\mathbf{x};\boldsymbol{\theta})$ to be a good approximation of the true distribution f_0, we want to find estimates for the model parameters $\boldsymbol{\theta}$ which most likely produce the measured data record $\mathbf{x}$. The likelihood function, which is to be maximized, is a function on the parameters $\boldsymbol{\theta}$

$$\mathcal{L}(\boldsymbol{\theta}) = f(\mathbf{x};\boldsymbol{\theta}) \ , \tag{S22}$$

and corresponds to the model f evaluated at the data points $\mathbf{x}$, where $\boldsymbol{\theta}$ are now parameters to be estimated. Thus, to define a likelihood function we need to make some assumption about the distribution of the data.

We now assume that the observations $\mathbf{x}$ are independent and their noise normally distributed. Thus, the approximating model becomes:

$$\begin{aligned} f(\mathbf{x};\boldsymbol{\theta}) &= f(x_1, x_2, \ldots, x_K;\boldsymbol{\theta}) = f(x_1;\boldsymbol{\theta}) f(x_2;\boldsymbol{\theta}) \ldots f(x_K;\boldsymbol{\theta}) \\ &= \left(\frac{1}{2\pi\sigma^2}\right)^{K/2} \exp\left(-\sum_{k=1}^{K} \frac{\epsilon_k^2}{2\sigma^2}\right) \ , \end{aligned} \tag{S23}$$

where ϵ_k are the residuals from the fitted model and σ^2 is an estimator of their variance. It is useful to work with logarithms because they are continuous and monotonous, thus the negative log-likelihood becomes:

$$-2\ln\big(\mathcal{L}(\boldsymbol{\theta})\big) = K\ln\left(2\pi\sigma^2\right) + \frac{\sum_{k=1}^{K}\epsilon_k^2}{\sigma^2} \approx K\ln\left(2\pi\sigma^2\right) + K \tag{S24}$$

$$-2\ln\big(\mathcal{L}(\boldsymbol{\theta})\big) \approx K\ln\left(\sigma^2\right) \ , \tag{S25}$$

where the approximation holds for large K and we have dropped the term $K\left(1 + \ln(2\pi)\right)$ because it is a constant offset [10, 12]. In our case, the data record $\mathbf{x}$ consists of a signal and a noise contribution. The latter is dominated by photon shot noise, which in the limit of many collected photons converges to a normal distribution. Using Eq. S21 as variance estimator we thus write:

$$K\ln\left(\sigma^2\right) = K\ln\left(\frac{\sum_{k=1}^{K}\left(x_k - \tilde{x}_k(\boldsymbol{\theta})\right)^2}{K - M - 1}\right) \ , \tag{S26}$$

where the function $\tilde{\mathbf{x}}(\boldsymbol{\theta})$ represents the chosen FID model for the set of parameters $\boldsymbol{\theta}$. Eq. (S26) represents the $G(\boldsymbol{\theta},\mathbf{x})$ term in Eqs. (8,9) in the main manuscript.

In our analysis, we fit the complex Fourier spectra instead of the time-domain FID traces. Denoting spectral quantities with a hat, we have for each complex spectral component

$$\hat{x}_j = \hat{a}_j + i\hat{b}_j = \sum_{k=1}^{K} x_k e^{-i2\pi kj} \ . \tag{S27}$$

The noise of the Fourier spectra is also normally distributed [8]. Since we assume the measurement points $\mathbf{x}$ to be independent, the noise in each the real $\hat{a}_j$ and imaginary $\hat{b}_j$ parts of the complex spectrum also follows a normal distribution, that is, $\hat{a}_j, \hat{b}_j \sim N\left(\mu = 0, \sigma_0^2\right)$ for $K \gg 1$. $\hat{a}_j$ and $\hat{b}_j$ are independent for $j < K/2$ [8].

In terms of the power spectrum, its components are $\hat{y}_j = |\hat{x}_j|^2 = \hat{a}_j^2 + \hat{b}_j^2$, meaning that their noise follows a chi-squared distribution with two degrees of freedom (χ_2^2). Therefore, the residues from the fitted model $\hat{\epsilon}_j = \hat{y}_j - \hat{\tilde{y}}_j(\boldsymbol{\theta})$ would follow a displaced chi-squared distribution $\chi_2^2\left(\frac{\hat{\epsilon}_j+\mu}{\sigma}\right) = \chi_2^2\left(\frac{\hat{\epsilon}_j+\sigma}{\sigma}\right)$, where we use standardized units and the fact that the expectation value μ for a χ_2^2 distribution equals its standard deviation σ. The model becomes:

$$\begin{aligned} f\left(\hat{\mathbf{y}}, \boldsymbol{\theta}\right) &= f\left(\hat{y}_1, \boldsymbol{\theta}\right) f\left(\hat{y}_2, \boldsymbol{\theta}\right) \dots f\left(\hat{y}_K, \boldsymbol{\theta}\right) \\ &= \chi_2^2\left(\frac{\hat{\epsilon}_1 + \mu}{\sigma}\right) \chi_2^2\left(\frac{\hat{\epsilon}_2 + \mu}{\sigma}\right) \dots \chi_2^2\left(\frac{\hat{\epsilon}_K + \mu}{\sigma}\right) \\ &= \left(\frac{1}{2\sigma}\right)^K \exp\left(-\frac{1}{2}\sum_{j=1}^{K} \frac{\hat{\epsilon}_j + \sigma}{\sigma}\right) = \left(\frac{1}{2\sigma}\right)^K e^{-K/2} \ , \end{aligned}$$

where we have taken into account that $\sum_{j=1}^{K} \hat{\epsilon}_j$ follows a normal distribution with zero mean (central-limit theorem for $K \gg 1$). Note the normalization factor $1/\sigma$ multiplying the χ_2^2 distributions such that their integrals are still unity. Taking the negative logarithm we find:

$$\begin{aligned} -2\ln\left(\mathcal{L}(\boldsymbol{\theta})\right) &= -2\ln\left(f\left(\hat{\mathbf{y}}, \boldsymbol{\theta}\right)\right) = 2K\ln\left(2\sigma\right) + K \\ &\approx K\ln\left(\sigma^2\right) \ , \end{aligned}$$

where we express the standard deviation as the square root of the variance estimator $\sigma = \sqrt{\sigma^2}$ and again dropped constant offsets. Using again Eq. S21, the negative log-likelihood function for the power spectrum becomes:

$$-2\ln\left(\mathcal{L}(\boldsymbol{\theta})\right) \approx K\ln\left(\sigma^2\right) = K\ln\left(\frac{\sum_{j=1}^{K}\left(\hat{y}_j - \hat{\tilde{y}}_j\left(\boldsymbol{\theta}\right)\right)^2}{K - M - 1}\right) \ , \tag{S28}$$

where $\hat{\tilde{\mathbf{y}}}\left(\boldsymbol{\theta}\right) = \{\hat{\tilde{y}}_j\}_{j=1\cdots K}$ represents the power spectrum of the chosen FID model $\tilde{\mathbf{x}}(\boldsymbol{\theta})$.

For a number D of independent datasets (*i.e.* FID time traces), we then write the combined

likelihood function for the spectral components and their square magnitude as:

$$\begin{aligned}
-2\ln\left(\mathcal{L}(\boldsymbol{\theta})\right) &= -2\ln\left(\prod_{d=1}^{D}\mathcal{L}_d(\boldsymbol{\theta})\right) = -2\ln\left(\prod_{d=1}^{D}\mathcal{L}_{d,re}(\boldsymbol{\theta})\mathcal{L}_{d,im}(\boldsymbol{\theta})\mathcal{L}_{d,psd}(\boldsymbol{\theta})\right) \\
&= -2\sum_{d=1}^{D}\ln\left(\mathcal{L}_{d,re}(\boldsymbol{\theta})\mathcal{L}_{d,im}(\boldsymbol{\theta})\mathcal{L}_{d,psd}(\boldsymbol{\theta})\right) \\
&= -2\sum_{d=1}^{D}\left(\ln\left(\mathcal{L}_{d,re}(\boldsymbol{\theta})\right)+\ln\left(\mathcal{L}_{d,im}(\boldsymbol{\theta})\right)+\ln\left(\mathcal{L}_{d,psd}(\boldsymbol{\theta})\right)\right) \\
&= K\sum_{d=1}^{D}\left(\ln\left(\sigma^2_{d,re}\right)+\ln\left(\sigma^2_{d,im}\right)+\ln\left(\sigma^2_{d,psd}\right)\right) \ ,
\end{aligned} \tag{S29}$$

where the subscripts $(\cdot)_{re}$, $(\cdot)_{im}$ and $(\cdot)_{psd}$ label the real, imaginary and magnitude-squared parts of the power spectra. Using Eq. S21 for the variance estimators, we finally write:

$$-2\ln\left(\mathcal{L}(\boldsymbol{\theta})\right) = -3DK\ln\left(K-M-1\right)+K\sum_{d=1}^{D}\sum_{ic}\ln\left(\Sigma_{d,ic}\right) \ , \tag{S30}$$

where $\Sigma_{d,ic}$ is the residual sum of squares for dataset d and $ic \in \{re, im, psd\}$. To find maximum likelihood estimates using the WIC we thus add Eq. S30 and Eq. S19.

3.2. Generalized Simulated Annealing (GSA)

GSA is a stochastic approach [16] that combines the original method of Classical Simulated Annealing (CSA) [17] and Fast Simulated Annealing (FSA) [18]. It has proven very useful to find global minima of complicated, multidimensional systems with large numbers of local minima such those found in quantum chemistry, genetics or non-linear time-series. Due to its statistical nature, local minima can be escaped much more easily than with steepest-descent or gradient methods [19]. The core idea is to combine importance sampling with an artificial temperature which is gradually decreased to simulate thermal noise. The algorithm uses a distorted Cauchy-Lorentz visiting distribution, to sample the parameter space [16]:

$$g_{q_v}\left(\Delta x_t\right) \propto \frac{T_{q_v}\left(t\right)^{-D/(3-q_v)}}{\left[1+(q_v-1)\frac{(\Delta x_t)^2}{\left[T_{q_v}(t)\right]^{2/(3-q_v)}}\right]^{1/(q_v-1)+(D-1)/2}} \ , \tag{S31}$$

where D is the number of dimensions (*i.e.* fit parameters). Eq. S31 is also known as a Tsallis distribution. Compared to the Boltzmann (CSA) or Cauchy (FSA) distributions, Eq. S31 has a longer tail and thereby combines a local search with frequent long jumps which facilitate detrapping from local minima. The shape of the distribution is controlled by the visiting parameter q_v, which also controls the cooling rate [16]:

$$T_{q_v}\left(t\right) = T_{q_v}\left(1\right)\frac{2^{q_v-1}-1}{(1+t)^{q_v-1}-1} \ . \tag{S32}$$

At (computational) time t and artificial visiting temperature $T_{q_v}(t)$, GSA thus requires the generation of random jumps Δx_t distributed according to Eq. S31. Here, we use an exact D-dimensional Tsallis random number generator [20]. A new configuration $x_t = x_{t-1} + \Delta x(t)$ is accepted with a probability [16]:

$$p_{q_a} = \min\left\{1, \left[1 - (1 - q_a)\, \beta \Delta E\right]^{1/(1-q_a)}\right\} , \tag{S33}$$

where q_a is an acceptance parameter and $\beta \propto T_a(t)^{-1}$ is inverse acceptance temperature. $T_a(t)$ is also controlled by q_v according to the cooling rate given by Eq. S32. Eq. S33 is a generalized Metropolis algorithm. Configurations with a lower cost ($\Delta E < 0$) are thus always accepted. For $q_a < 0$, zero acceptance probability is assigned to the cases where [16]:

$$1 - (1 - q_a)\, \beta \Delta E < 0 , \tag{S34}$$

meaning that ΔE must be smaller than $\beta / (1 - q_a)$ for hill-climbing to occur. The choice of $q_a < 0$ in the argument of the power-law acceptance function (Eq. S33) was originally suggested because it favors lower energies. However, alternative choices of p_{q_a} for $q_a > 1$ have also been proposed [21]. Setting $q_v = q_a = 1$ recovers CSA, and $q_v = 2$, $q_a = 1$ recovers FSA [16]. As $T \to 0$ (or $t \to \infty$), the algorithm becomes a steepest-descent method [19].

For our calculations, we selected $q_v = 2.7$, $q_a = -1$ and a large initial acceptance temperature $T_a(1) \sim 10^3$ to facilitate the escape from local minima. A technique to accelerate convergence is to set:

$$T_a(t) \to \frac{T_a(t)}{t} , \tag{S35}$$

which is equivalent to a monotonously decreasing $q_a \propto t^{-1}$ [19]. It is worth noting that the computational times associated with the visiting and acceptance temperatures need not to be equal. To improve convergence towards physically-relevant configurations, we modified the acceptance probability $p_{q_a} \to p'_{q_a}$ using a step-function:

$$p'_{q_a} = p_s \cdot p_{q_a} \tag{S36}$$

$$p_s = \begin{cases} 1 & \text{if } d_{ij} > r_{cc}\ \forall i \neq j \\ 0 & \text{otherwise} \end{cases} , \tag{S37}$$

where d_{ij} is the estimated spatial distance between any two spins i and j at any computational time t, and $r_{cc} = 0.154\,\text{nm}$ is the diamond bond-length. In other words, we reject any proposed nuclear-spin configurations where any two spins are closer to each other than the smallest ^{13}C -^{13}C distance in a diamond crystal.

We also assigned an individual visiting temperature $T_{q_v,m}$ to each fit parameter θ_m in order to account for their different scales/units, and set their initial values large enough such that their entire search intervals were covered at $t = 0$ (see Sec. 3 3.4). Furthermore, we

observed better results when normalizing the visiting and acceptance times to a few hundred Monte Carlo steps. Specifically, we used $t_v = t/400$ and $t_a = t/200$. This re-scaling of the computational time effectively helps to more widely explore the parameter space at each temperature step. We also observed better convergence when updating one parameter at a time instead of the whole parameter set.

For a given number n of spins, we run the algorithm starting from $\sim 10^2 - 10^3$ initial random spin configurations, where each configuration is drawn by randomly selecting n points within a radius of 2.5 nm around the coordinate origin. We ran the algorithm over 5000 iterations and took the best result. As a guide, a single run (1 initial spin configuration) of the algorithm for $n \approx 20$ took ~ 3 h.

It is worth noting that the performance of GSA, and in general of annealing algorithms, is problem dependent and therefore some parameter tuning is usually required. Our selections for q_v and q_a lie within ranges where good performance has been reported [16].

3.3. Estimation of uncertainties

As mentioned in the main text, we simultaneously fitted all spectra. To get an estimate for the uncertainties in the fit parameters $\boldsymbol{\theta}$, we resampled the data using a bootstrapping protocol [22]. We calculated the mean and standard deviation of the fit residues (green dots in Fig. 4b,c in the main text) for the Re[FFT] and Im[FFT] parts of each spectrum. We then used the resulting deviations to generate normally-distributed noise and thereby created a set of $P = 100$ new, artificial datasets. Each newly generated dataset (set of 4 spectra) was minimized again (using a steepest-descent search) and the original-dataset solution as starting point. We thereby generated a set $\theta_{m,p} \equiv \{\theta_m\}_p$ of estimates for each fit parameter θ_m, where $p = 1 \ldots P$. Finally, we calculated the fit results as:

$$\begin{aligned} \bar{\theta}_m &= \langle \theta_{m,p} \rangle = \frac{1}{P} \sum_{p=1}^{P} \theta_{m,p} \\ \sigma(\theta_m) &= \left(\frac{1}{P-1} \sum_{p=1}^{P} \left(\theta_{m,p} - \bar{\theta}_m \right)^2 \right)^{1/2} \end{aligned} \tag{S38}$$

3.4. Selection of parameter intervals

To start our minimization routines, we chose random starting values from a pre-defined interval for each parameter.

For $a_{||,i}$, we defined the interval by looking at the spectral support across all power spectra and set it to $2\pi \times [2\,(f_0 - f_k) \ldots 2\,(f_0 + f_k)]$, where f_0 is the central-peak frequency (assumed to be around the bare Larmor frequency) and we selected f_k such that all visible peaks in the power spectra where covered. Specifically, we selected $f_k = 4.5$ kHz and $f_k = 4$ kHz for dataset 1 and 2, respectively.

For $a_{\perp,i}$, we defined a sufficiently large interval to cover the minimum and maximum observable couplings. Specifically, we chose $2\pi \times [0.0 \ldots 80\,\text{kHz}]$ and $2\pi \times [0.0 \ldots 70\,\text{kHz}]$ for dataset 1 and dataset 2, respectively.

We initialized ϕ_i in the interval $[0.0 \ldots 360\,°]$, but constrained it to the interval $[-90.0 \ldots 450\,°]$ to avoid phase-wrapping with values close to 0° or 360°. Finally, we assumed starting intervals for the global parameters as $p_0 \in [0.3 \ldots 1.0]$, $\Gamma_0 \in [0 \ldots (12\,\text{ms})^{-1}]$ and $t_\ell \in [0 \ldots t_\text{s}]$.

3.5. Global parameters p_0, $T^*_{2,\text{n}}$ and t_ℓ

On top of the measurement-induced dephasing with rate $\Gamma_\beta = \beta^2/(4t_\text{s})$, nuclear spins are also subject to intrinsic dephasing with rate $\Gamma_0 = (T^*_{2,\text{n}})^{-1}$ due to T_2^* relaxation and further relaxation with rate Γ_optical caused by the optical readout. Since T_2^* dephasing in our experiment is dominated by fluctuations in the bias field, we can treat Γ_0 as a global fit parameter. The readout-induced dephasing is explained as follows: owing to our off-resonant optical readout and re-initialization scheme, the NV center undergoes stochastic spin-state, electronic-state and potentially charge-state transitions during optical illumination before reaching the polarized $m_S = 0$ state [2, 3]. The residual hyperfine interaction during laser readout causes an additional dephasing with rate [1]:

$$\Gamma_\text{optical} = \frac{\left(a_{||} t_\ell\right)^2}{2t_\text{s}}$$

where t_ℓ is a phenomenological time constant that is roughly of the duration of the laser pulse. t_ℓ is not correlated with the ^{13}C environment [2, 3] and is a global free fit parameter in our maximum likelihood estimation. The total decay rate is then the sum of all contributions

$$\Gamma = \Gamma_\beta + \Gamma_0 + \Gamma_\text{optical} = \frac{\beta^2}{4t_\text{s}} + \frac{1}{T^*_{2,\text{n}}} + \frac{a_{||}^2 t_\ell^2}{2t_\text{s}} \tag{S39}$$

The third parameter, the initial nuclear polarization p_0, in principle differs for every ^{13}C nuclei, because the polarization rate is proportional to $a_\perp$. However, because we repeat the polarization transfer for typically $> 10^3$ cycles, we expect that the polarization of all nuclei within a resonant slice become saturated. (This level is reduced for pairs or clusters of spins with near-identical $(a_{||}, a_\perp)$ coupling constants, however, not many such pairs are expected for our dilute ^{13}C concentration). This assumption is supported by the fact that spectra show little change in peak intensities once the number of cycles exceeds 10^3 (see Ref. 1, Extended Data Fig. 5) and is consistent with similar models using cross-relaxation induced polarization (CRIP) [23]. Therefore, we assume p_0 to become homogeneous within our sensitive slices and treat it as a global parameter.

4. INTERNUCLEAR COUPLINGS

Continuous weak measurements can be regarded as the equivalent of inductive detection in conventional NMR. In this way, they capture the full evolution of the nuclear environment,

including potential nuclear spin-spin interactions. Such nuclear spin-spin interactions lead to additional peaks in the spectra, which can interfere with our minimization strategy. To illustrate the effect of nuclear spin-spin interactions and analyze whether they affect our present experiment, we performed a density matrix simulation with $n = 3$ randomly positioned nuclear spins using a Master equation approach.

Fig. S4 shows the simulated weak measurement spectrum for nuclear spins with inter-nuclear coupling constants $g_{12} = 634.79\,\mathrm{Hz}$, $g_{13} = 1.17\,\mathrm{Hz}$, and $g_{23} = 22.05\,\mathrm{Hz}$. The coupling constants are chosen arbitrarily. All splittings (orange arrows) within the FFT resolution ($t_\mathrm{s} = 8\ \mu\mathrm{s}$) are visible, illustrating how weak measurement-spectroscopy gives access to the whole dynamics. The direct consequence is that weak measurement spectroscopy can potentially yield very complex spectra, similar to inductively-detected Fourier NMR spectroscopy.

To estimate whether such couplings are potentially present in our experimental spectra, we computed the average number of spin pairs yielding resolvable couplings within our spectral resolution given by $\Gamma = \max\{1/\left(Kt_\mathrm{s}\right), \Gamma_0\}$. The FFT resolution is 156 Hz and 110 Hz for NV1 and NV2, and the estimated nuclear linewidth is $\Gamma_0 = 1/T^*_{2,\mathrm{n}} = 86 \pm 7\,\mathrm{Hz}$ for NV1 and $120 \pm 11\,\mathrm{Hz}$ for NV2, limited by fluctuations in the magnetic bias field. We generate 10^4 random ^{13}C nuclear spin configurations within the sensitive volumes of NV1 and NV2 (Fig. 5a,d in the main text), respectively, and count the number of pairs with couplings larger than Γ. Considering the worst-case scenario (neglecting the influence of measurement and readout-induced dephasing), we find on average 1.14 ± 1.06 (69% probability that at least one spin-pair is present) and 2.14 ± 1.43 (89% probability) spin-pairs for NV1 and NV2 , respectively. Thus, although the presence of one or two spin pairs is likely, this will not affect the majority of peaks observed in the spectrum. Moreover, since the two spins forming a pair will have very similar hyperfine coupling constants, their peaks will be spectrally close, and the peak splitting may not be resolved because of peak overlap.

As a control, we have inspected the reconstructed spatial positions of the mapped nuclei and computed their relative spatial distances. We find an average distance between the closest pairs of 0.212 nm and 0.178 nm for dataset 1 and 2, respectively. This corresponds to homonuclear couplings of 544 Hz and 645 Hz. However, due to the inversion symmetry of the diamond crystal, each recovered nuclear position has a 50% probability to be mirrored along the coordinate origin.

5. SUPPLEMENTARY TABLES

Figure 4b	
NV center	1
B field	201.29 mT
Electronic $T_{2,e}$ (CPMG)	216 μs
NV initialization laser pulse	1.5 μs
NV readout laser pulse	1.5 μs
Weak measurement CPMG pulses	$\{4, 8, 12, 16\}$
Weak measurement CPMG duration t_β	$\{0.930, 1.860, 2.790, 3.720\}$ μs
Number of weak measurements K	800
Sampling period t_s	8.0 μs
Duration of LR-NOVEL pulse t_{novel}	30 μs
Ramp amplitude of LR-NOVEL pulse	10 %
$\pi/2$ rotation RF pulse	10.903 μs
Number of polarization repetitions M	1200
Total integration time	10 h, equal to 2.5 h for each t_β

TABLE S1. Measurement parameters for Figure 4b. A detailed pulse diagram is given the Extended Data Fig. 1b of Ref. 1.

Figure 4c	
NV center	2
B field	188.89 mT
Electronic $T_{2,e}$ (CPMG)	80 μs
NV initialization laser pulse	1.5 μs
NV readout laser pulse	1.5 μs
Weak measurement CPMG pulses	$\{12, 16, 20, 24\}$
Weak measurement CPMG duration t_β	$\{2.966, 3.956, 4.944, 5.932\}$ μs
Number of weak measurements K	800
Sampling period t_s	11.48 μs
Duration of LR-NOVEL pulse t_{novel}	25 μs
Ramp amplitude of LR-NOVEL pulse	10 %
$\pi/2$ rotation RF pulse	20.520 μs
Number of polarization repetitions M	1600
Total integration time	45.73 h, equal to 11.43 h for each t_β

TABLE S2. Measurement parameters for Figure 4c. The parameters for the FID shown in *Fig. 4a* are those for $t_\beta = 4.944$ μs. A detailed pulse diagram is given the Extended Data Fig. 1b of Ref. 1.

^{13}C	$a_{\|\|}\,(2\pi \cdot \text{kHz})$	$a_{\perp}\,(2\pi \cdot \text{kHz})$	$r\,(\text{nm})$	$\theta\,(\text{deg})$	$\phi\,(\text{deg})$	δV (Å^3)
1	-8.14 ± 0.12	36.40 ± 4.66	0.89 ± 0.03	60.88 ± 0.77	137.20 ± 6.21	0.26
2	-5.47 ± 0.08	22.80 ± 3.97	1.04 ± 0.05	61.38 ± 1.16	322.71 ± 17.27	2.22
3	-5.10 ± 0.06	9.07 ± 2.76	1.32 ± 0.11	68.77 ± 3.82	312.55 ± 30.21	29.09*
4	-3.41 ± 0.03	21.53 ± 2.85	1.07 ± 0.04	59.18 ± 0.59	13.96 ± 10.49	0.87
5	-3.18 ± 0.03	9.43 ± 3.02	1.38 ± 0.16	64.27 ± 3.67	59.99 ± 12.13	20.29*
6	-1.91 ± 0.04	7.54 ± 2.08	1.51 ± 0.14	62.05 ± 2.46	250.42 ± 19.13	25.72*
7	-1.81 ± 0.03	16.95 ± 2.26	1.17 ± 0.05	57.77 ± 0.42	345.77 ± 13.86	1.58
8	-0.69 ± 0.03	18.40 ± 2.67	1.15 ± 0.06	55.83 ± 0.17	48.00 ± 8.45	1.16
9	-0.69 ± 0.04	36.80 ± 3.03	0.91 ± 0.03	55.28 ± 0.05	118.14 ± 12.01	0.26
10	-0.27 ± 0.02	17.63 ± 2.89	1.17 ± 0.07	55.18 ± 0.09	160.57 ± 7.13	1.39
11	-0.15 ± 0.05	23.36 ± 3.61	1.07 ± 0.06	54.93 ± 0.07	36.75 ± 10.71	1.39
12	0.12 ± 0.01	9.83 ± 2.77	1.45 ± 0.19	54.34 ± 0.33	325.24 ± 15.78	31.24*
13	0.46 ± 0.05	23.67 ± 3.17	1.07 ± 0.05	54.17 ± 0.10	137.58 ± 9.80	0.94
14	1.02 ± 0.03	12.03 ± 2.12	1.35 ± 0.09	52.19 ± 0.52	262.02 ± 12.74	4.83
15	1.78 ± 0.05	3.20 ± 0.82	2.11 ± 0.16	38.11 ± 4.31	312.18 ± 25.23	101.10*
16	2.76 ± 0.13	75.59 ± 3.41	0.72 ± 0.01	53.68 ± 0.07	179.94 ± 12.90	0.04
17	2.81 ± 0.08	59.09 ± 3.50	0.79 ± 0.02	53.36 ± 0.09	130.90 ± 9.42	0.07
18	3.53 ± 0.07	30.62 ± 3.58	0.99 ± 0.04	51.34 ± 0.42	229.59 ± 13.11	0.80
19	4.56 ± 0.08	8.24 ± 4.01	1.49 ± 0.49	37.57 ± 10.99	251.81 ± 40.95	710.58*
20	4.83 ± 0.09	8.18 ± 3.47	1.52 ± 0.35	36.46 ± 9.19	290.20 ± 40.89	409.71*

TABLE S3. ^{13}C nuclear spins localization results for NV 1. Fit results for global parameters are $p_0 = 0.66 \pm 0.03$, $t_\ell = 1.65 \pm 0.25\ \mu$s and $\Gamma_0 = 86.27 \pm 7.43\,$Hz corresponding to an intrinsic $T^*_{2,\text{n}}$ (upper bound) of $11.68 \pm 1.03\,$ms. Errors for $a_{||}$, $a_\perp$ and ϕ are calculated by bootstrapping. Errors for r, ϑ, ϕ and the global parameters are calculated using Monte Carlo error propagation. The estimated uncertainty volume δV (also depicted in Fig. 5c) is given in cubic Angstrom. * denote spins whose uncertainty volume is larger than the volume per carbon atom in the diamond lattice (5.69 Å^3).

^{13}C	$a_{\|\|}\,(2\pi\cdot\text{kHz})$	$a_{\perp}\,(2\pi\cdot\text{kHz})$	r (nm)	θ (deg)	ϕ (deg)	δV (Å^3)
1	-5.60 ± 0.02	4.06 ± 0.60	1.45 ± 0.02	77.72 ± 1.47	164.47 ± 8.24	0.74
2	-4.78 ± 0.20	57.29 ± 4.17	0.78 ± 0.02	57.10 ± 0.20	163.35 ± 6.66	0.07
3	-3.92 ± 0.08	39.69 ± 2.56	0.88 ± 0.02	57.52 ± 0.19	289.33 ± 5.14	0.06
4	-1.50 ± 0.04	1.55 ± 0.32	2.17 ± 0.06	74.12 ± 2.29	310.91 ± 31.43	27.20*
5	-1.26 ± 0.01	8.34 ± 1.10	1.47 ± 0.06	58.98 ± 0.56	188.47 ± 5.48	1.18
6	-0.68 ± 0.17	49.71 ± 5.63	0.83 ± 0.03	55.13 ± 0.11	99.38 ± 6.51	0.20
7	-0.38 ± 0.02	24.89 ± 1.74	1.04 ± 0.02	55.17 ± 0.04	207.35 ± 5.99	0.14
8	-0.17 ± 0.01	9.33 ± 1.50	1.45 ± 0.08	55.28 ± 0.10	320.92 ± 6.50	2.27
9	-0.10 ± 0.01	8.97 ± 1.62	1.47 ± 0.09	55.08 ± 0.08	292.63 ± 6.46	3.09
10	-0.03 ± 0.01	22.18 ± 2.10	1.08 ± 0.03	54.77 ± 0.01	312.39 ± 3.27	0.16
11	0.14 ± 0.01	9.66 ± 1.24	1.44 ± 0.06	54.31 ± 0.06	112.11 ± 8.04	1.72
12	0.17 ± 0.02	2.30 ± 0.97	2.39 ± 0.80	51.95 ± 3.95	22.60 ± 13.98	816.44*
13	0.43 ± 0.38	66.69 ± 5.07	0.75 ± 0.02	54.55 ± 0.17	255.44 ± 9.13	0.09
14	0.73 ± 0.01	8.06 ± 1.18	1.54 ± 0.08	52.04 ± 0.43	203.91 ± 3.66	1.35
15	0.86 ± 0.00	10.77 ± 1.11	1.39 ± 0.05	52.39 ± 0.25	16.24 ± 2.98	0.38
16	0.86 ± 0.10	42.23 ± 4.07	0.88 ± 0.03	54.14 ± 0.09	116.56 ± 5.53	0.15
17	1.19 ± 0.01	3.16 ± 0.42	2.12 ± 0.10	43.55 ± 1.53	351.77 ± 14.40	13.72*
18	1.52 ± 0.02	3.82 ± 0.66	1.99 ± 0.12	42.88 ± 2.14	344.02 ± 11.21	15.60*
19	1.83 ± 0.01	5.22 ± 0.66	1.79 ± 0.08	44.33 ± 1.34	85.48 ± 7.82	3.92
20	2.06 ± 0.42	65.55 ± 4.99	0.76 ± 0.02	53.83 ± 0.20	182.83 ± 9.30	0.10
21	2.79 ± 0.02	2.98 ± 0.47	2.06 ± 0.07	30.22 ± 2.86	225.82 ± 14.11	16.62*
22	2.86 ± 0.15	45.03 ± 3.90	0.86 ± 0.03	52.88 ± 0.19	358.76 ± 13.09	0.28
23	3.17 ± 0.02	3.46 ± 0.60	1.97 ± 0.08	30.51 ± 3.18	49.28 ± 13.58	17.30*
24	4.20 ± 0.12	41.97 ± 3.04	0.88 ± 0.02	51.81 ± 0.23	262.86 ± 4.70	0.08
25	4.81 ± 0.09	39.67 ± 3.00	0.90 ± 0.02	51.18 ± 0.28	278.17 ± 4.68	0.09
26	5.17 ± 0.27	66.03 ± 4.48	0.76 ± 0.02	52.45 ± 0.20	328.24 ± 8.23	0.07
27	6.53 ± 0.04	19.60 ± 2.48	1.15 ± 0.05	44.86 ± 1.28	120.54 ± 7.46	0.99
28	6.61 ± 0.02	9.53 ± 2.63	1.45 ± 0.11	34.65 ± 5.09	305.34 ± 13.18	19.93*
29	6.72 ± 0.04	7.04 ± 2.28	1.54 ± 0.14	29.16 ± 6.46	186.44 ± 17.03	47.63*

TABLE S4. ^{13}C nuclear spins localization results for NV 2. Fit results for global parameters are $p_0 = 0.43\pm0.02$, $t_\ell = 1.86\pm0.24\,\mu$s and $\Gamma_0 = 119.94\pm10.62\,$Hz corresponding to an intrinsic $T_{2,\mathrm{n}}^*$ (upper bound) of $8.4\pm0.76\,$ms. Errors for $a_{||}$, $a_\perp$ and ϕ are calculated by bootstrapping. Errors for r, ϑ, ϕ and the global parameters are calculated using Monte Carlo error propagation. The estimated uncertainty volume δV (also depicted in Fig. 5f) is given in cubic Angstrom. * denote spins whose uncertainty volume is larger than the volume per carbon atom in the diamond lattice (5.69 Å^3).

6. SUPPLEMENTARY FIGURES

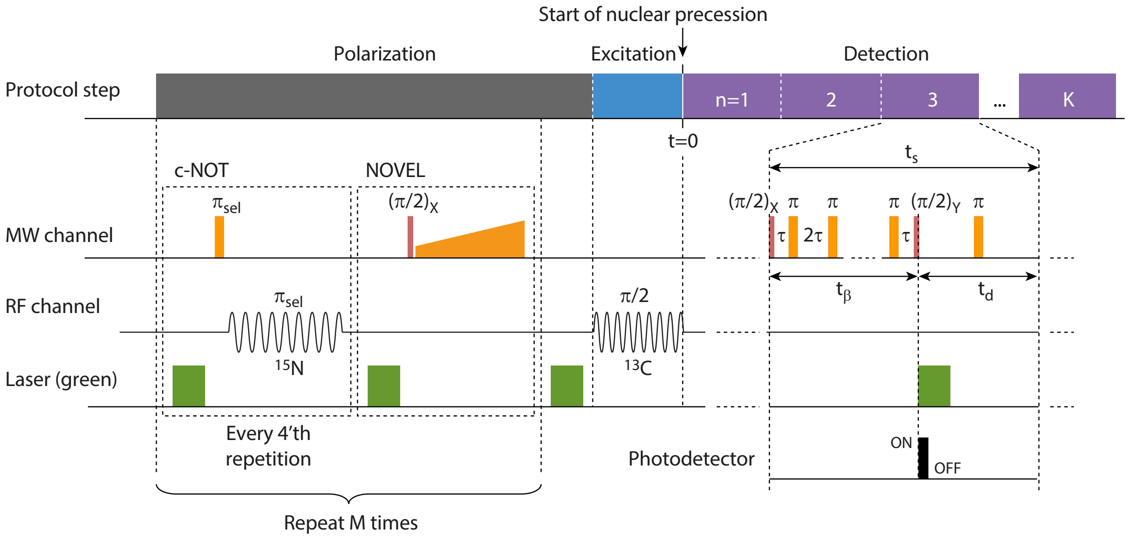

FIG. S1. **Experimental sequence.** Horizontal axis is time. Yellow and red blocks are microwave (MW) π and $\pi/2$ pulses, respectively, that act on the lower-energy ($m_S = 0$ to -1) transition of the electronic spin. Black sinusoidal curves are radio-frequency (RF) pulses acting on the ^{15}N (frequency $\sim$ 0.86 MHz) and ^{13}C (frequency $\sim$ 2.1 MHz) nuclear spins. Green blocks are laser pulses with typical duration 1.5 μs. After each laser pulse we include a delay of $\sim$ 1 μs to allow the NV center to decay to the electronic ground state. Our protocol proceeds in three steps: Polarization, excitation and detection. The polarization step consists of $M \sim 10^3$ repetitions of the NOVEL sequence [6, 24]. The NOVEL sequence consists of a $(\pi/2)_X$ rotation followed by a spin-lock along Y of duration t_{novel}. We use a linear amplitude ramp of the spin-lock pulse of $\sim$ 10% to improve robustness of the polarization transfer. Every four NOVEL cycles we add a c-NOT gate to polarize the ^{15}N nuclear spin to improve the polarization transfer efficiency. The c-NOT gate consists of a selective MW π pulse (conditional on the ^{15}N spin state) followed by a selective RF π pulse (conditional to the electronic spin state) [25]. The excitation step consists of a single, non-selective RF pulse on the ^{13}C nuclear spins. The detection step consists of a train of $K \sim 10^3$ weak measurement readouts [1, 8, 26, 27]. Each weak measurement block consists of an XY8 sequence [28] of between 4-24 MW π pulses, sandwiched by two MW $\pi/2$ pulses that have a relative phase shift of 90° [29]. The delay between π pulses (2τ) is adjusted to the inverse of the expected ^{13}C Larmor frequency f, $\tau \approx 1/(4f)$. The XY8 sequence is followed by a delay time t_d. During the delay, the electronic spin state is read out by applying a green laser pulse and turning on the photo-detector for $\sim$ 300 ns. An additional MW π pulse at $t_d/2$ is used to average the hyperfine interaction [29]. The sampling time $t_s = t_\beta + t_d$ is adjusted, via the delay time, to match an $(j + 0.25)$ multiple of the expected ^{13}C Larmor precession period, where j is an integer and the 0.25 implements a time-proportional phase increment (TPPI) [30]. All measurement parameters are collected in Tables S1 and S2.

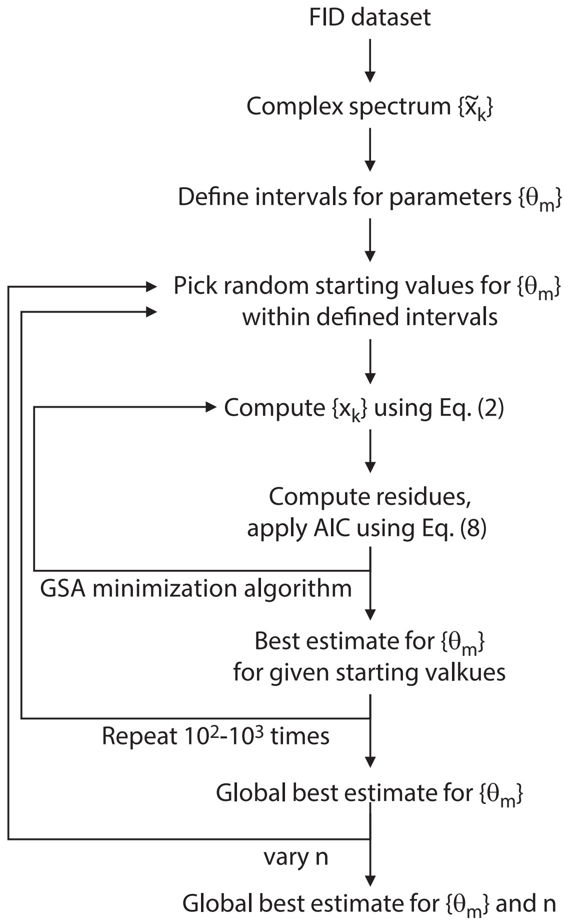

FIG. S2. **Flow diagram for maximum likelihood estimation** as discussed in Section VI of the main manuscript and Section 4 of the Supplementary Information.

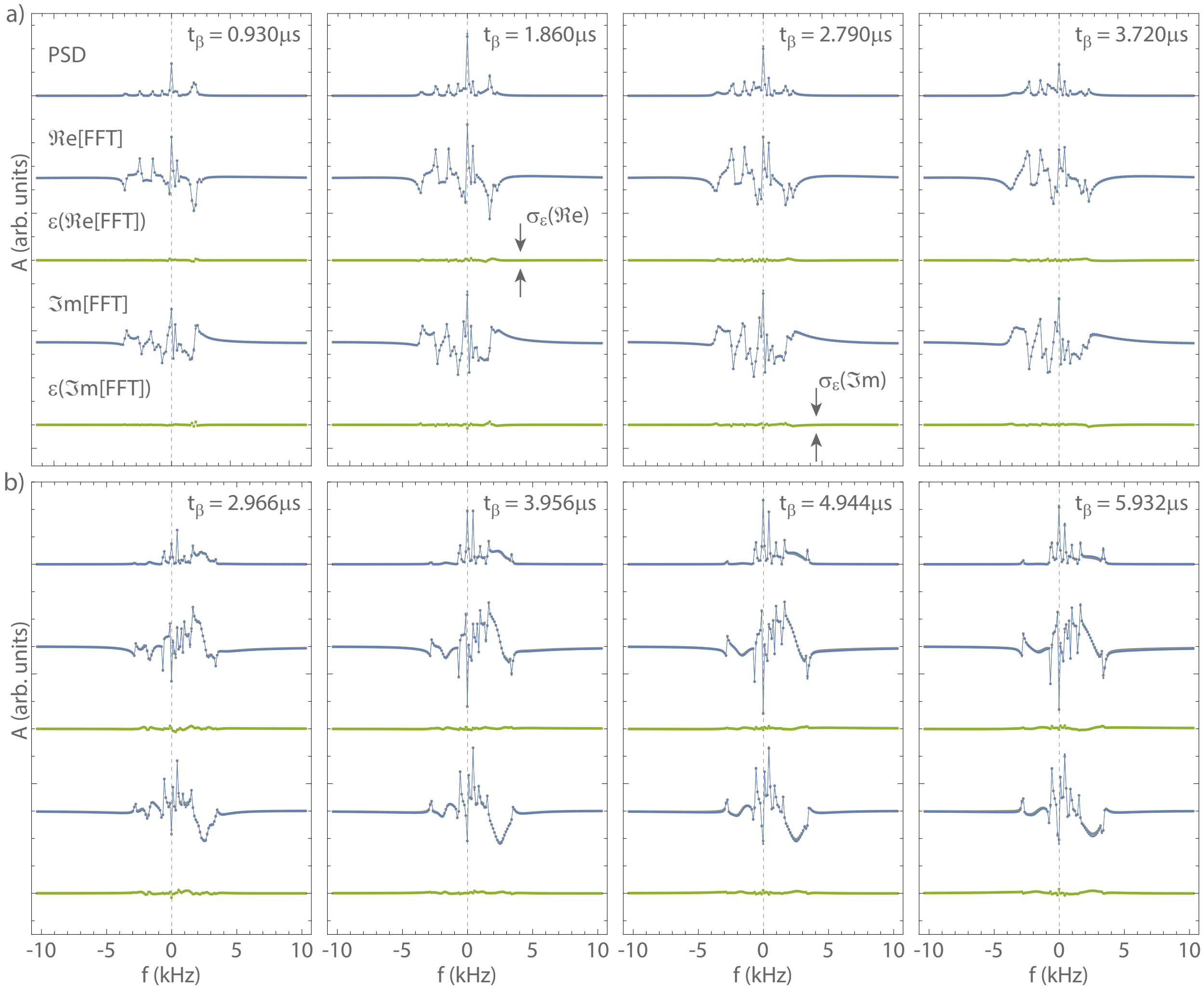

FIG. S3. **Second-order analytical model and numerical density-matrix simulations.** Complex Fourier spectra of the ^{13}C environment (NV2) for a series of interaction times t_β. Shown are from top to bottom (vertically offset for clarity): power spectrum (PSD), real part of the complex spectrum (Re[FFT]), fit residues for Re[FFT], imaginary part of the complex spectrum (Im[FFT]), and fit residues for Im[FFT]. The horizontal axis shows the spectral shift relative to the ^{13}C Larmor frequency. To illustrate the agreement between our analytical FID model (Eq. 2 in the main text) and the numerical density-matrix simulations, we take the measurement parameters (Table S2) and the best-fit parameters obtained for NV2 and simulate the resulting set of spectra (blue traces) using the density matrix formalism. The gray traces show the corresponding spectra obtained using our analytical model. The negligible residues (green traces), well smaller than those in Figs.4b,c in the main text, highlight the excellent match between the density-matrix simulations and our second-order analytical model.

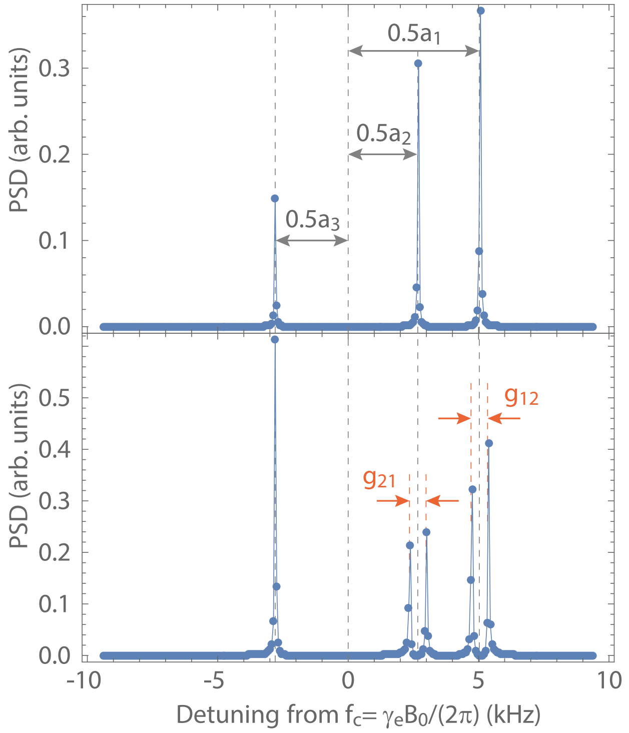

FIG. S4. **Simulation of weak measurement spectroscopy with non-vanishing internuclear couplings.** We consider three ^{13}C nuclear spins with the arbitrary hyperfine couplings $a_{||} = \{10.056, 5.338, -5.593\}$ kHz and $a_{\perp} = \{16.863, 13.990, 9.359\}$ kHz and nuclear ^{13}C -^{13}C couplings $\{g_{12} = 634.79, g_{13} = 1.17, g_{23} = 22.05\}$ Hz. The bias field is $B_0 = 202\,\mathrm{mT}$. We simulate a time trace of $K = 2,000$ weak measurements ($t_\beta = 0.465$ μs) at a sampling time $t_\mathrm{s} = 8$ μs and plot the power spectrum (blue dots) against the detuning from the detection frequency $f_\mathrm{c} = 1/(2\tau)$, where τ is the spacing between π pulses (Fig. 2b in the main text). In the absence of ^{13}C -^{13}C couplings (top panel), we observe three peaks associated with the three nuclear spins. The offset from f_c is proportional to the parallel hyperfine coupling constant (grey arrows). In contrast, for non-vanishing internuclear couplings (bottom panel), splittings proportional to g_{ij} appear but only those within the spectral resolution $\delta f = 1/(Kt_\mathrm{s}) = 156.25\,\mathrm{Hz}$ set by the Fourier limit of the time trace are resolved (orange arrows).

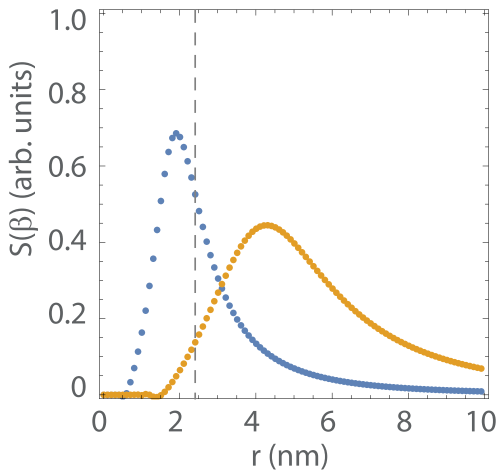

FIG. S5. **Sensitive slice extrapolated for single ^{1}H detection.** Sensitive slice $\mathcal{S}(\beta)$ as a function of distance r for ^{13}C and ^{1}H detection. The blue curve (^{13}C) uses the experimental settings for dataset 2 and $t_{\beta} = 5.932\,\mu$s. The orange curve (^{1}H) uses $t_{\beta} = T_{2,\mathrm{e}} = 50\,\mu$s and $T^{*}_{2,\mathrm{n}} = 10\,$ms. The vertical dashed-line at $r = 2.4\,$nm indicates the most distant nucleus (^{13}C #12) that we measured in dataset 2.

[1] K. S. Cujia, J. M. Boss, K. Herb, J. Zopes, and C. L. Degen, Tracking the precession of single nuclear spins by weak measurements, Nature **571**, 230 (2019).

[2] G. Waldherr, J. Beck, M. Steiner, O. Neumann, A. Gali, T. H. Frauenheim, F. Jelezko, and J. Wrachtrup, Dark states of single nitrogen-vacancy centers in diamond unraveled by single shot nmr, Phys. Rev. Lett. **106**, 157601 (2011), 1012.5017.

[3] S. Dhomkar, J. Henshaw, H. Jayakumar, and C. A. Meriles, Long-term data storage in diamond, Science Advances **2**, e1600911 (2016), 1610.09022.

[4] T. Unden, N. Tomek, T. Weggler, F. Frank, P. London, J. Zopes, C. L. Degen, N. Raatz, J. Meijer, H. Watanabe, K. M. Itoh, M. B. Plenio, B. Naydenov, and F. Jelezko, Coherent control of solid state nuclear spin nano-ensembles, npj Quantum Information **4**, 39 (2018).

[5] J. Randall, C. E. Bradley, F. V. van der Gronden, A. Galicia, M. H. Abobeih, M. Markham, D. J. Twitchen, F. Machado, N. Y. Yao, and T. H. Taminiau, Observation of a many-body-localized discrete time crystal with a programmable spin-based quantum simulator, Science **0**, eabk0603 (2021).

[6] A. Henstra, P. Dirksen, J. Schmidt, and W. Wenckebach, Nuclear spin orientation via electron spin locking (NOVEL), Journal of Magnetic Resonance **77**, 389 (1988).

[7] T. Villazon, P. W. Claeys, M. Pandey, A. Polkovnikov, and A. Chandran, Persistent dark states in anisotropic central spin models, Scientific Reports **10**, 10.1038/s41598-020-73015-1 (2020).

[8] J. M. Boss, K. S. Cujia, J. Zopes, and C. L. Degen, Quantum sensing with arbitrary frequency resolution, Science **356**, 837 (2017).

[9] K. P. Burnham and D. R. Anderson, *Model Selection and Multimodel Inference* (Springer-Verlag New York, 2002).

[10] H. Akaike, A new look at the statistical model identification, IEEE Transactions on Automatic Control **19**, 716 (1974).

[11] G. Schwarz, Estimating the dimension of a model, Ann. Statist. **6**, 461 (1978).

[12] C. M. Hurvich and C.-L. Tsai, Regression and time series model selection in small samples, Biometrika **76**, 297 (1989).

[13] S. Rinke and P. Sibbertsen, Information criteria for nonlinear time series models, Studies in Nonlinear Dynamics and Econometrics **20**, 325 (2016).

[14] T.-J. Wu and A. Sepulveda, The weighted average information criterion for order selection in time series and regression models, Statistics and Probability Letters **39**, 1 (1998).

[15] A. McQuarrie, R. Shumway, and C.-L. Tsai, The model selection criterion aicu, Statistics and Probability Letters **34**, 285 (1997).

[16] C. Tsallis and D. A. Stariolo, Generalized simulated annealing, Physica A: Statistical Mechanics and its Applications **233**, 395 (1996).

[17] S. Kirkpatrick, C. D. Gelatt, and M. P. Vecchi, Optimization by simulated annealing, Science

220, 671 (1983), https://science.sciencemag.org/content/220/4598/671.full.pdf.
[18] H. Szu and R. Hartley, Fast simulated annealing, Physics Letters A **122**, 157 (1987).
[19] Y. Xiang, D. Sun, W. Fan, and X. Gong, Generalized simulated annealing algorithm and its application to the thomson model, Physics Letters A **233**, 216 (1997).
[20] T. Schanze, An exact d-dimensional tsallis random number generator for generalized simulated annealing, Computer Physics Communications **175**, 708 (2006).
[21] I. Andricioaei and J. E. Straub, Generalized simulated annealing algorithms using tsallis statistics: Application to conformational optimization of a tetrapeptide, Phys. Rev. E **53**, R3055 (1996).
[22] B. Efron, Bootstrap Methods: Another Look at the Jackknife, Ann. Statist. **7**, 1 (1979).
[23] D. A. Broadway, J. P. Tetienne, A. Stacey, J. A. Wood, D. A. Simpson, L. T. Hall, and L. C. L. Hollenberg, Quantum probe hyperpolarisation of molecular nuclear spins, Nature Communications **9**, 1246 (2018).
[24] P. London, J. Scheuer, J. M. Cai, I. Schwarz, A. Retzker, M. B. Plenio, M. Katagiri, T. Teraji, S. Koizumi, J. Isoya, R. Fischer, L. P. Mcguinness, B. Naydenov, and F. Jelezko, Detecting and polarizing nuclear spins with double resonance on a single electron spin, Phys. Rev. Lett. **111**, 067601 (2013).
[25] T. Rosskopf, J. Zopes, J. M. Boss, and C. L. Degen, A quantum spectrum analyzer enhanced by a nuclear spin memory, NPJ Quantum Information **3**, 33 (2017).
[26] S. Schmitt, T. Gefen, F. M. Sturner, T. Unden, G. Wolff, C. Muller, J. Scheuer, B. Naydenov, M. Markham, S. Pezzagna, J. Meijer, I. Schwarz, M. Plenio, A. Retzker, L. P. McGuinness, and F. Jelezko, Submillihertz magnetic spectroscopy performed with a nanoscale quantum sensor, Science **356**, 832 (2017).
[27] D. R. Glenn, D. B. Bucher, J. Lee, M. D. Lukin, H. Park, and R. L. Walsworth, High-resolution magnetic resonance spectroscopy using a solid-state spin sensor, Nature **555**, 351 (2018).
[28] T. Gullion, D. B. Baker, and M. S. Conradi, New, compensated carr-purcell sequences, J. Magn. Res. **89**, 479 (1990).
[29] J. M. Boss, K. Chang, J. Armijo, K. Cujia, T. Rosskopf, J. R. Maze, and C. L. Degen, One- and two-dimensional nuclear magnetic resonance spectroscopy with a diamond quantum sensor, Phys. Rev. Lett. **116**, 197601 (2016).
[30] R. R. Ernst, G. Bodenhausen, and A. Wokaun, Principles of nuclear magnetic resonance in one and two dimensions, (International Series of Monographs on Chemistry, Clarendon Press, 1990).
